# Supplementary material for: Simulating Finite-Time Isothermal Processes with Superconducting Quantum Circuits
Source: Entropy (Basel). 2021 Mar 16;23(3):353. doi: 10.3390/e23030353 (PMC8002232; doi:10.3390/e23030353)

This note provides the details of the fully quantum simulation for the two-step isothermal process on ibmqx2. The simulation results of the excited state population are shown in the following table.

		Exact	ibmqx2				
Short time $\delta\tau = 0.5$	$p_e(0)$	0.26894	0.25671	0.25391	0.26196	0.27026	0.25232
	$p_e(\delta\tau)$	0.22180	0.24097	0.24365	0.24731	0.24133	0.24036
	$p_e(2\delta\tau)$	0.17242	0.22266	0.23108	0.22375	0.22644	0.22791
Long time $\delta\tau = 10$	$p_e(0)$	0.26894	0.25586	0.27185	0.26086	0.26306	0.26978
	$p_e(\delta\tau)$	0.18243	0.21887	0.20728	0.21240	0.21228	0.20911
	$p_e(2\delta\tau)$	0.11920	0.15527	0.15051	0.14832	0.14417	0.15536

The results and the circuits of all steps on ibmqx2 are shown as follows. In the result, the index of the qubits is organized as 1, 2, 3, 4 and 5. The qubit 3 represents the system qubit, while the rest are the ancillary qubits. The measurement is performed on the system qubit only, with other qubits undetected. In the histograms, the state 0 on qubit (1, 2, 4, 5) is marked for simplicity.

Short-time dynamics with $\delta\tau = 0.5$

The population of the system state is measured at $t = 0, \delta\tau, 2\delta\tau$. The circuit implemented on ibmqx2 at different steps is given as follows.

1. Prepare the initial state at $t = 0$.

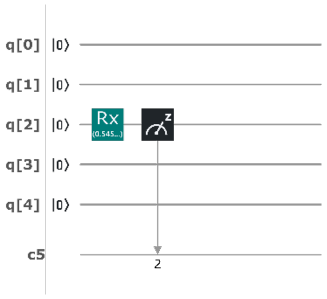

Initially, we need to prepare the initial state. The circuit is implemented on ibmqx2 for continuous 40960 shots in five runs. The excited state population in the five runs is $p_e(0) = 0.25671, 0.25391, 0.26196, 0.27026, 0.25232$. The raw data was obtained on April 16th, 2020.

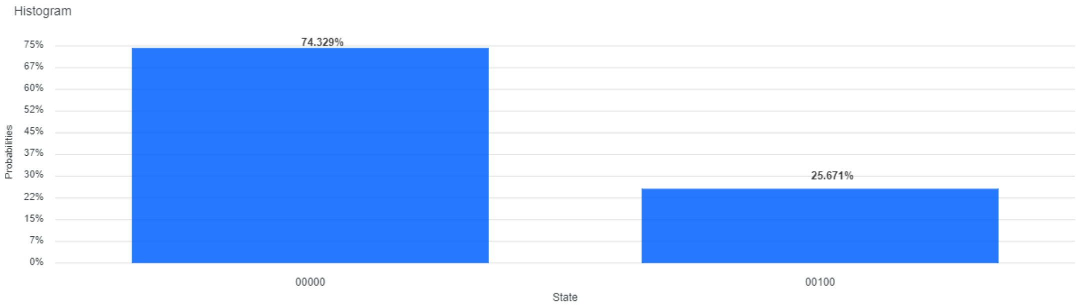
Histogram
75%
67%
60%
52%
45%
37%
30%
22%
15%
7%
0%
Probabilities
74.329%
25.671%
00000
00100
State

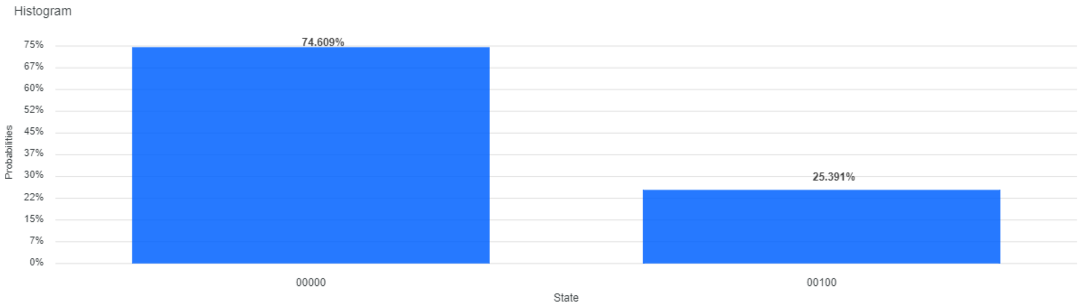
Histogram
75%
67%
60%
52%
45%
37%
30%
22%
15%
7%
0%
Probabilities
74.609%
25.391%
00000
00100
State

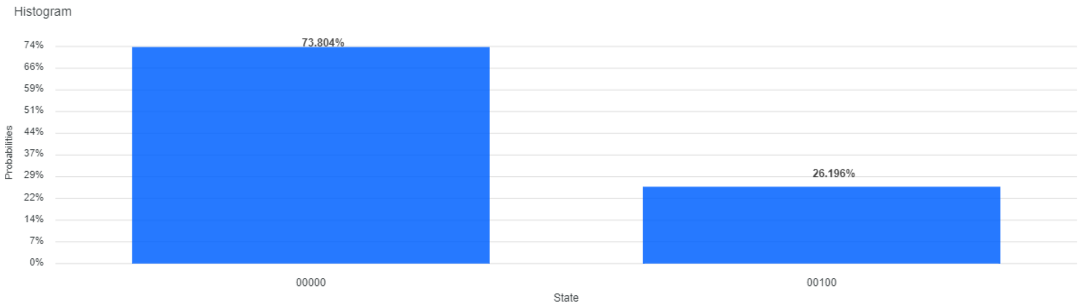
Histogram
74%
66%
59%
51%
44%
37%
29%
22%
14%
7%
0%
Probabilities
73.804%
26.196%
00000
00100
State

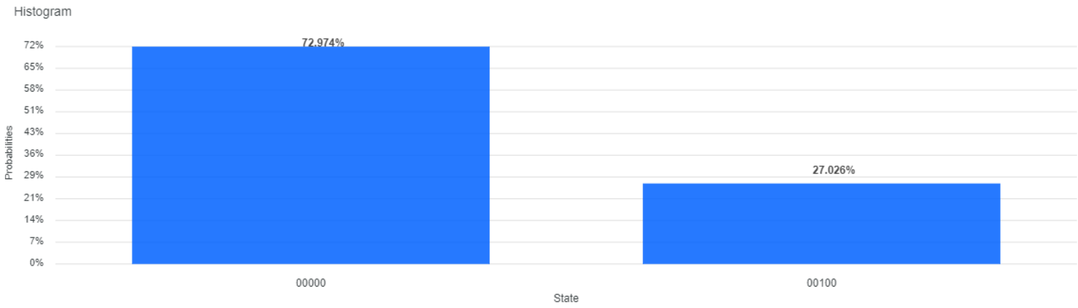
Histogram
72%
65%
58%
51%
43%
36%
29%
21%
14%
7%
0%
Probabilities
72.974%
27.026%
00000
00100
State

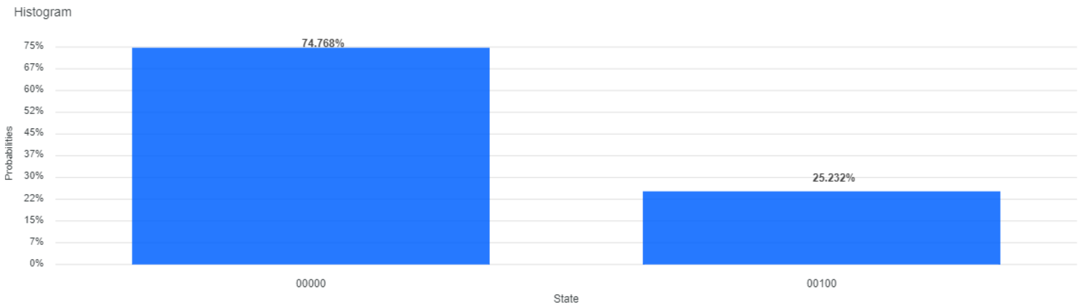
Histogram
75%
67%
60%
52%
45%
37%
30%
22%
15%
7%
0%
Probabilities
74.768%
25.232%
00000
00100
State

2. The first step $t = \delta\tau$.

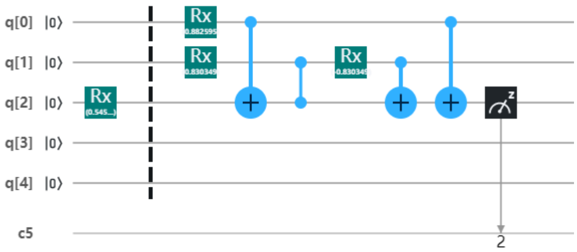

The circuit is implemented on ibmqx2 for continuous 40960 shots in five runs. The excited state population in the five runs is $p_e(\delta\tau) = 0.24097, 0.24365, 0.24731, 0.24133, 0.24036$. The raw data was obtained on April 16th, 2020.

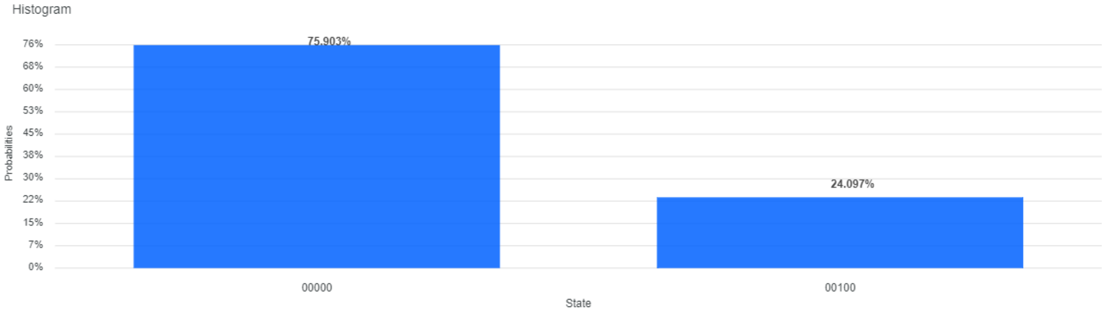

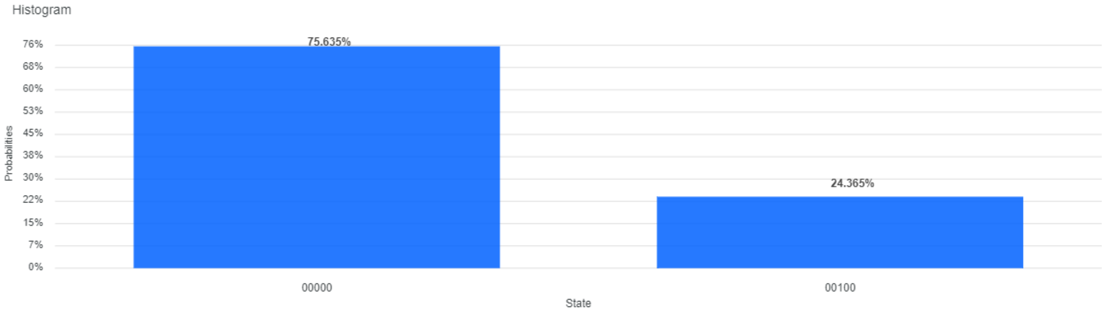

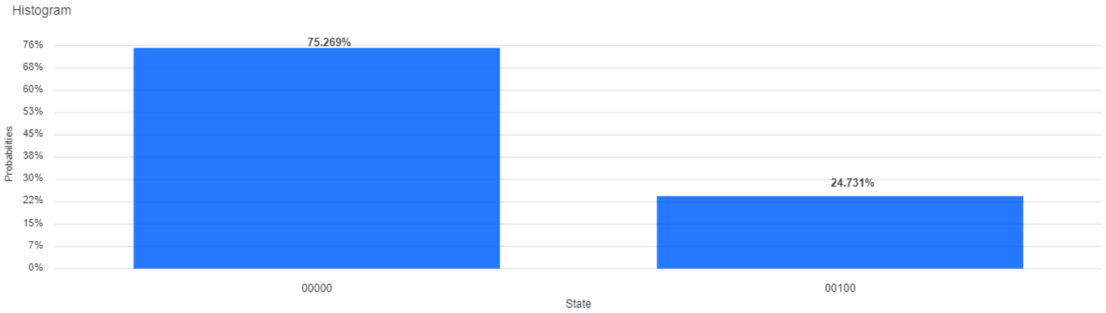

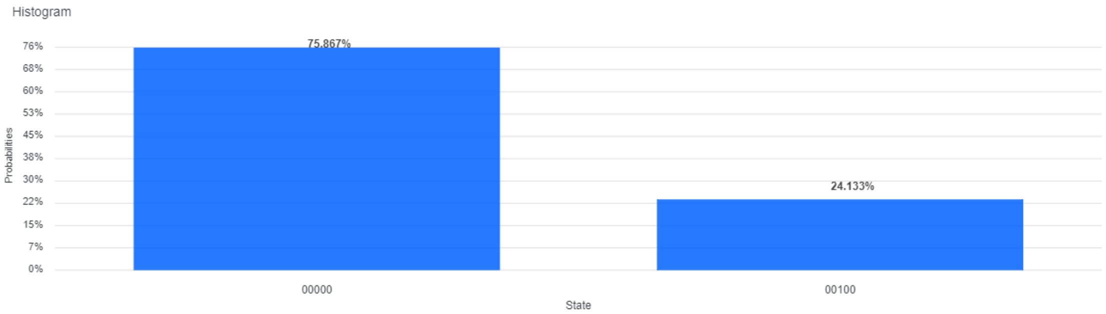

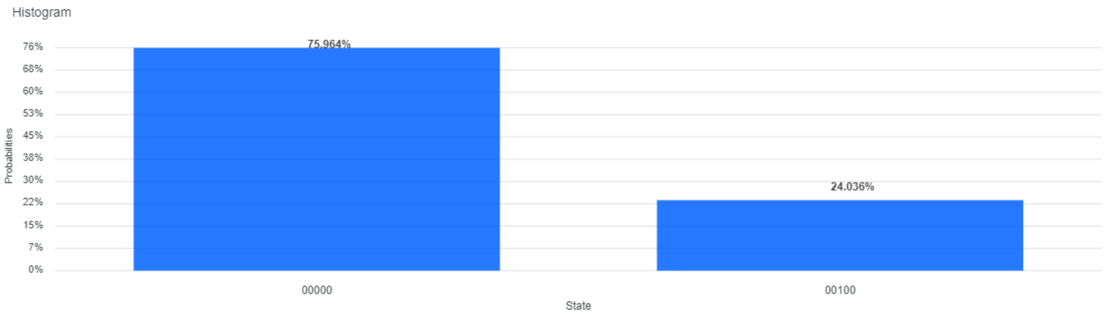

3. The second step $t = 2\delta\tau$.

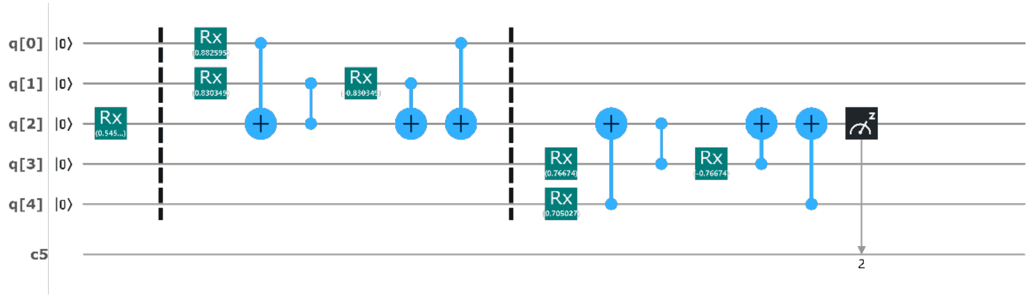

The circuit is implemented on ibmqx2 for continuous 40960 shots in five runs. The excited state population in the five runs is $p_e(2\delta\tau) = 0.22266, 0.23108, 0.22375, 0.22644, 0.22791$. The raw data was obtained on April 16th, 2020.

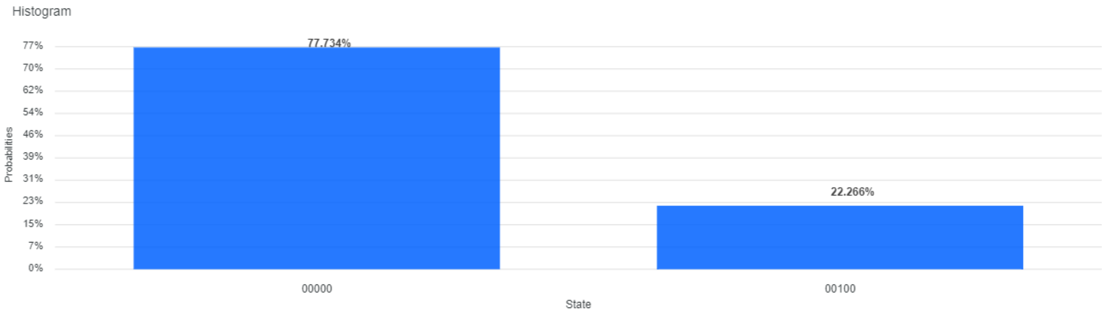

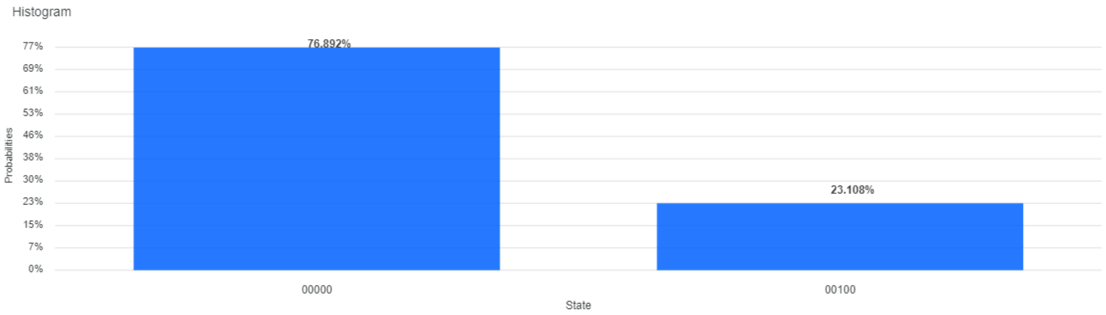
Histogram
Probabilities
77%
69%
61%
53%
46%
38%
30%
23%
15%
7%
0%
76.892%
23.108%
00000
00100
State

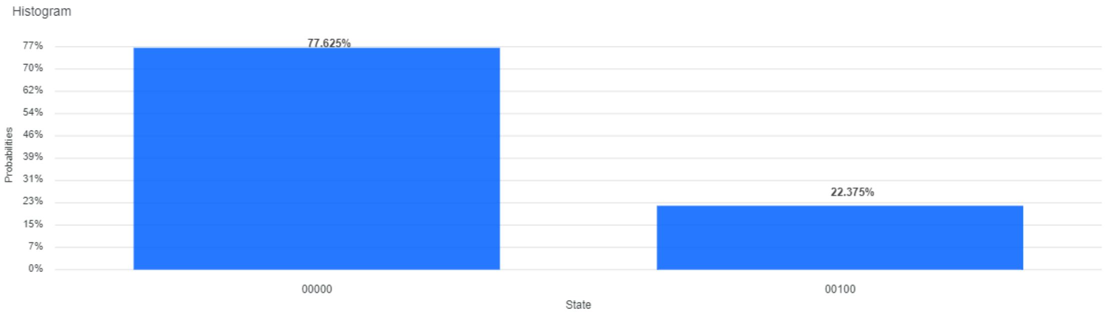
Histogram
Probabilities
77%
70%
62%
54%
46%
39%
31%
23%
15%
7%
0%
77.625%
22.375%
00000
00100
State

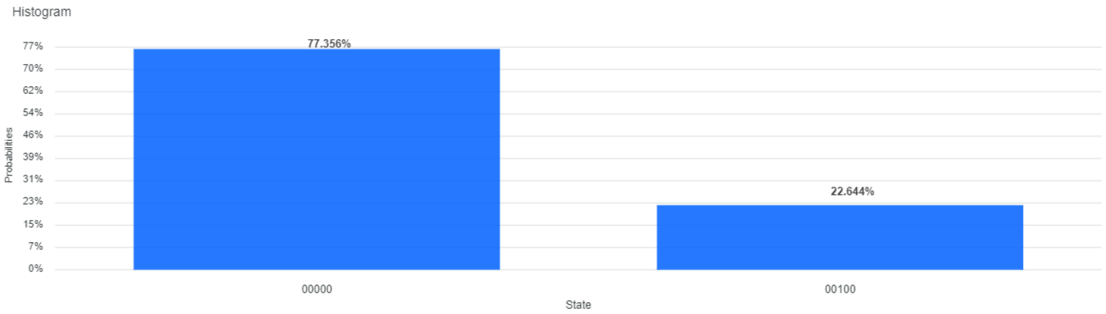
Histogram
Probabilities
77%
70%
62%
54%
46%
39%
31%
23%
15%
7%
0%
77.356%
22.644%
00000
00100
State

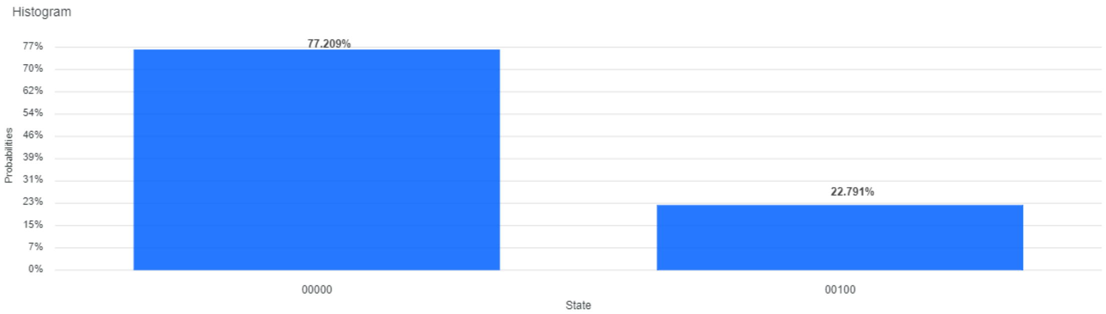
Histogram
Probabilities
77%
70%
62%
54%
46%
39%
31%
23%
15%
7%
0%
77.209%
22.791%
00000
00100
State

Long-time dynamics with $\delta\tau = 10$

1. Prepare the initial state $t = 0$.

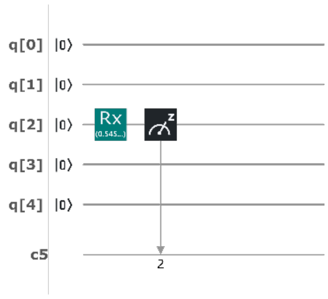

The circuit to prepare the initial state is the same, which is implemented on ibmqx2 for continuous 40960 shots in five runs. The excited state population in the five runs is $p_e(0) = 0.25586$, $0.27185, 0.26086, 0.26306, 0.26978$. The raw data was obtained on April 16th, 2020.

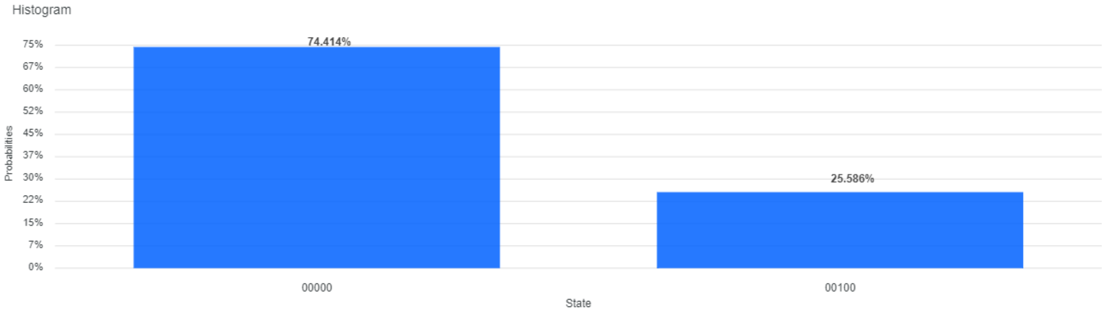

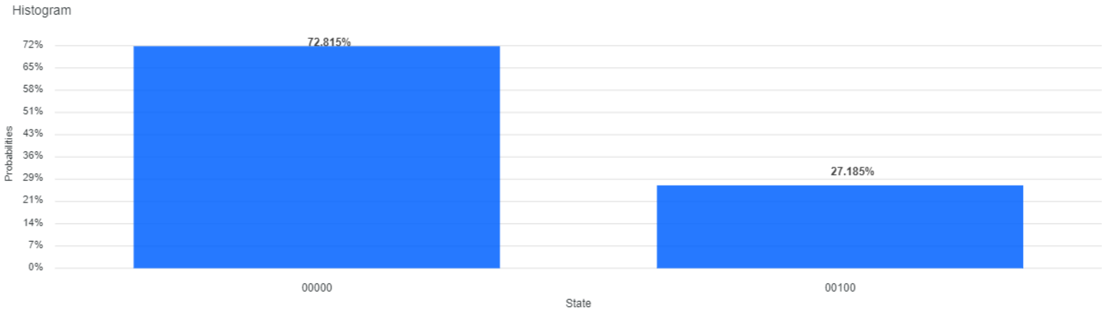

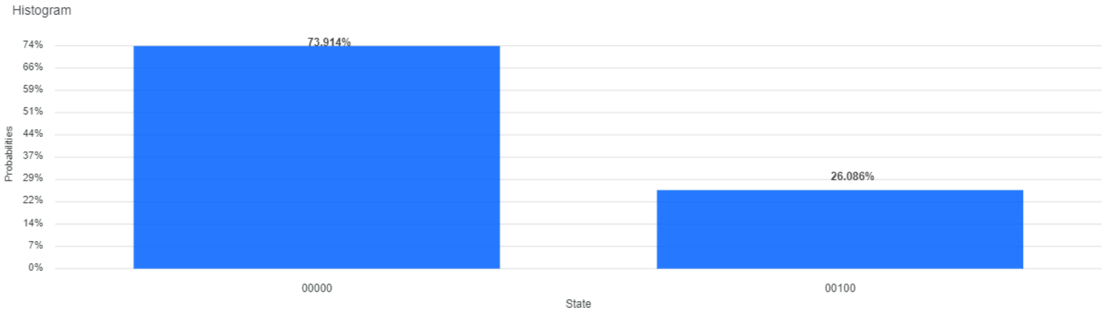

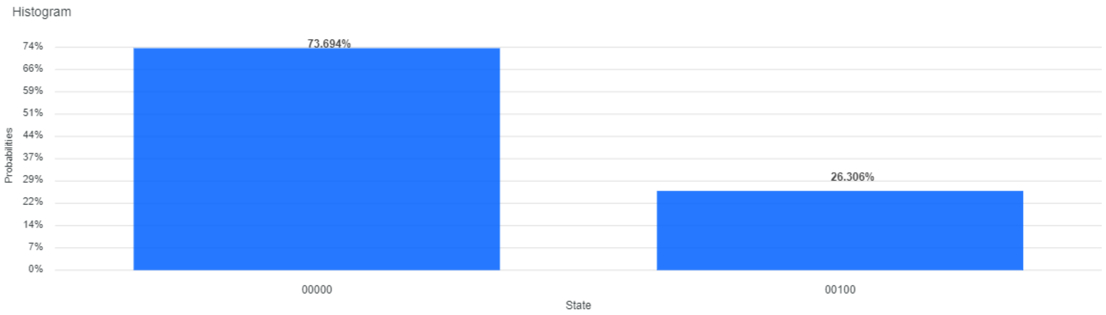

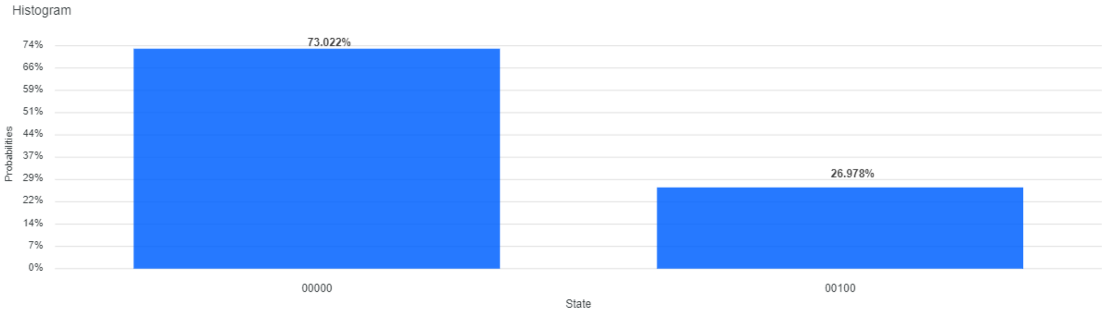

2. The first step $t = \delta\tau$.

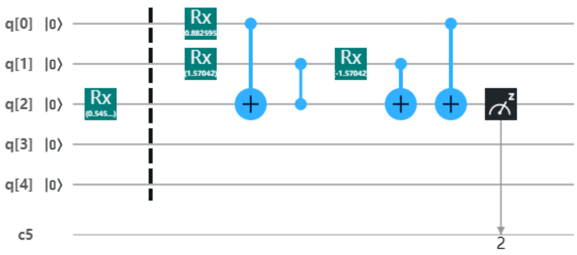

The circuit is implemented on ibmqx2 for continuous 40960 shots in five runs. The excited state population in the five runs is $p_e(\delta\tau) = 0.21887, 0.20728, 0.21240, 0.21228, 0.20911$. The raw data was obtained on April 16th, 2020.

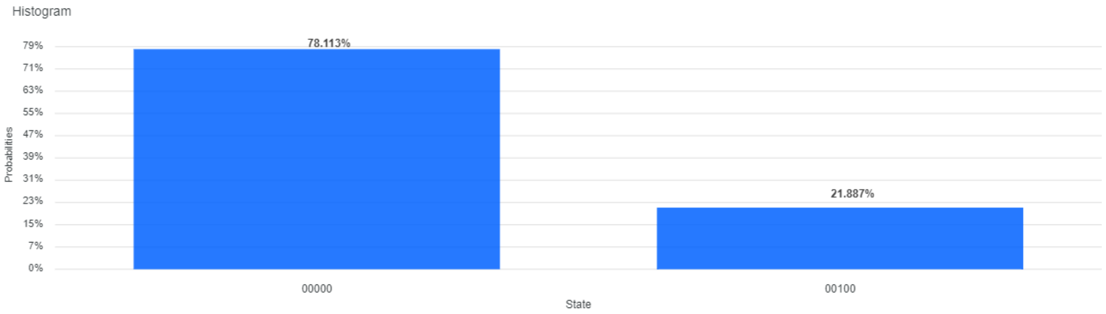

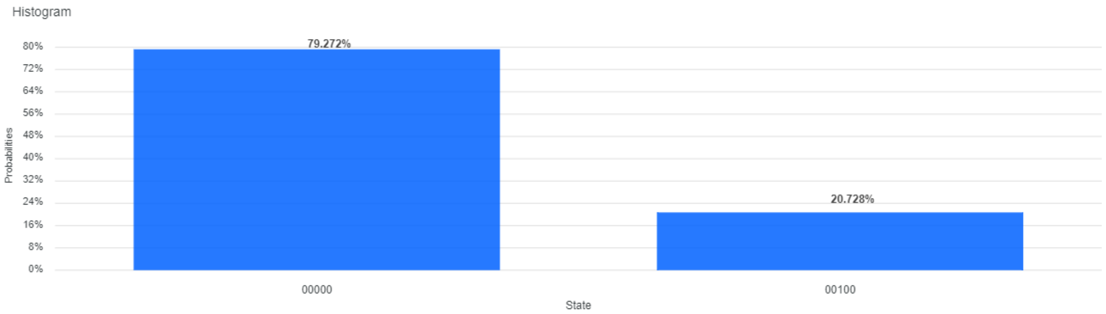

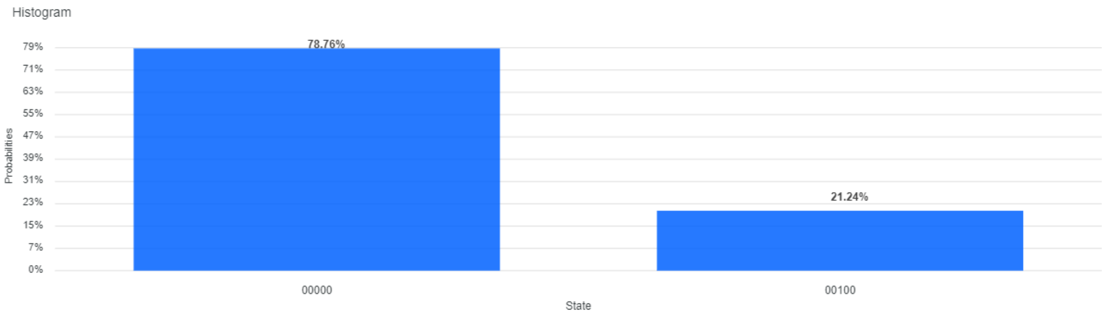

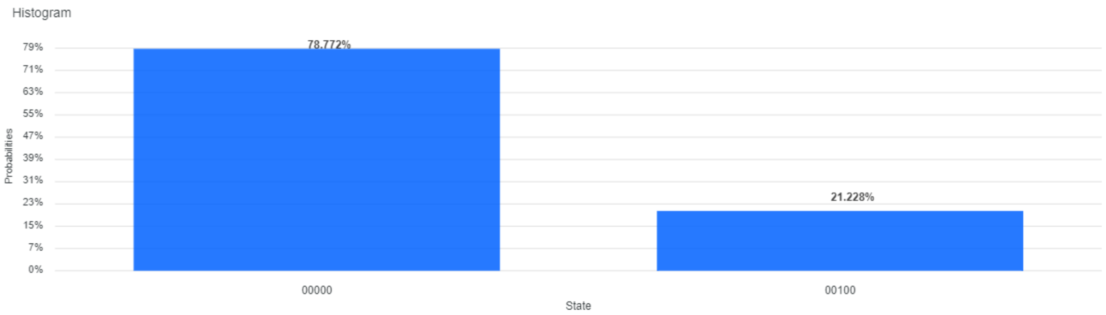

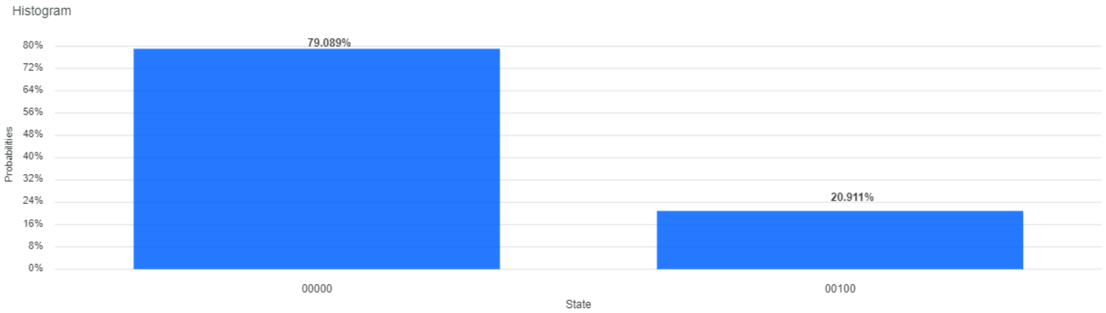

3. The second step $t = 2\delta\tau$.

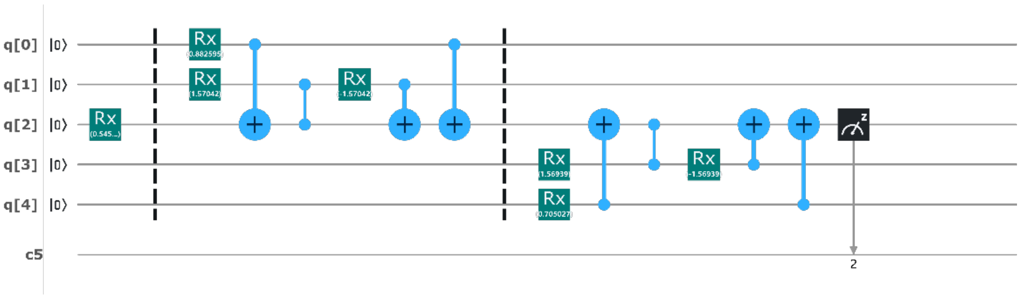

The circuit is implemented on ibmqx2 for continuous 40960 shots in five runs. The excited state

population in the five runs is $p_e(2\delta\tau) = 0.15527, 0.15051, 0.14832, 0.14417, 0.15536$. The raw data was obtained on April 16th, 2020.

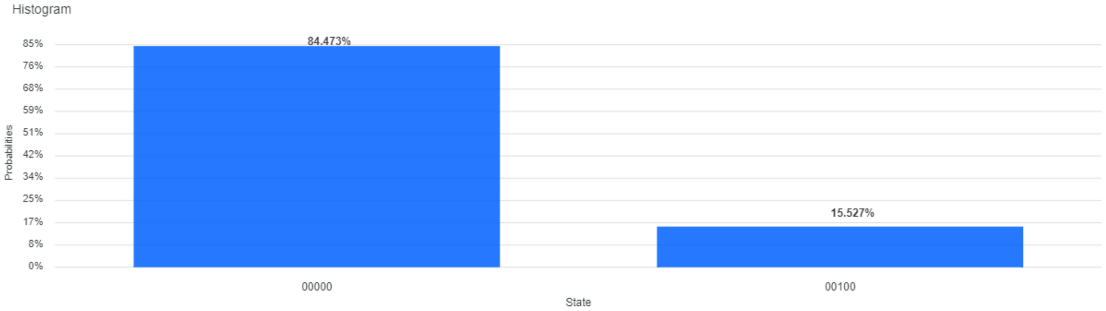

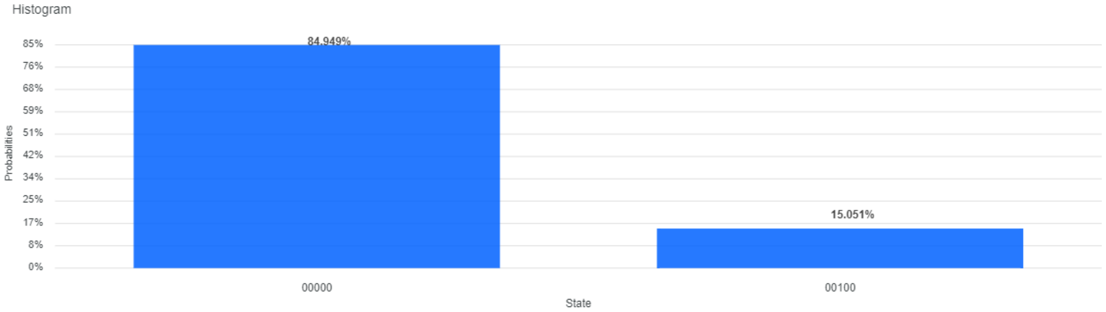

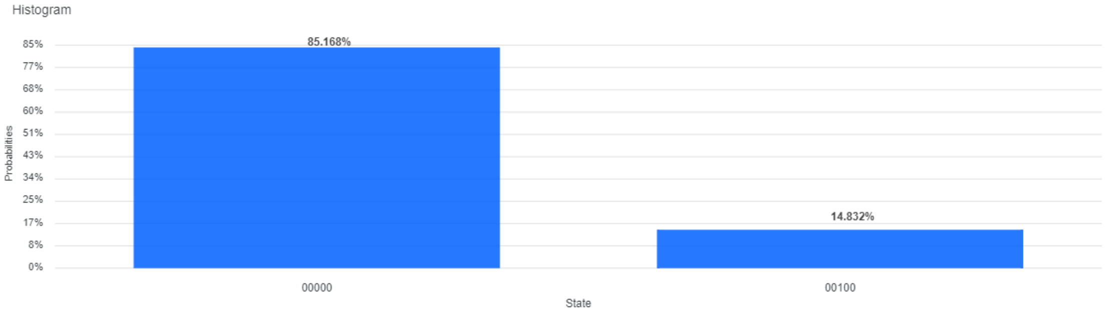

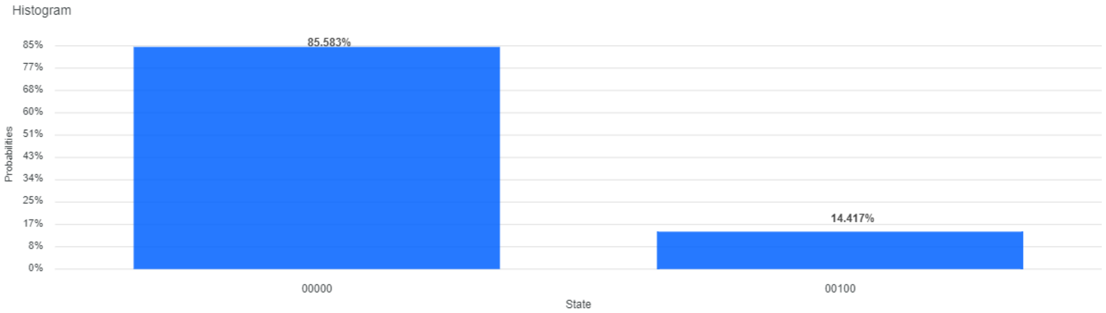

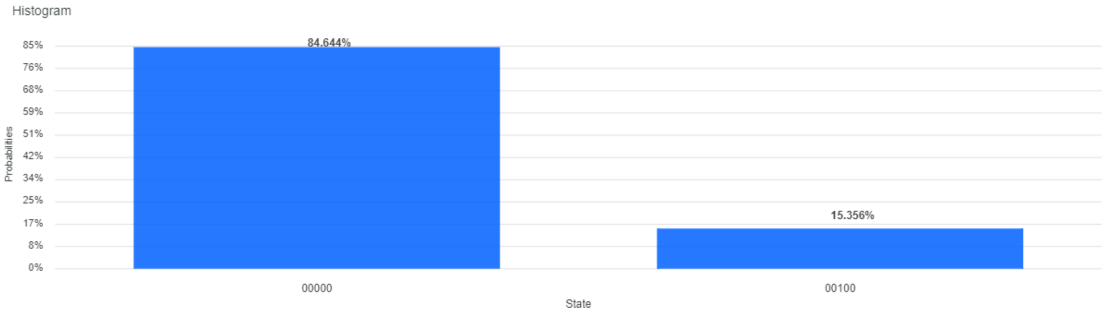

Supplement: Supplementary file 1 [file entropy-23-00353-s001.zip › fully_quantum_simulation_results.pdf]
