# Supplementary material for: Simulating Finite-Time Isothermal Processes with Superconducting Quantum Circuits
Source: Entropy (Basel). 2021 Mar 16;23(3):353. doi: 10.3390/e23030353 (PMC8002232; doi:10.3390/e23030353)
Supplement: Supplementary file 1 [file entropy-23-00353-s001.zip › hybrid_simulation_results.pdf]

This note provides the simulation details of the hybrid simulation on ibmqx2. We show the ibmqx2 results of the excited state population in the following table, which is compared to the exact numerical results.

	Sub-channel	Exact	ibmqx2		Sub-channel	Exact	ibmqx2
$\delta\tau = 0.5$, $N = 2$	Initial	0.26894	0.25391	$\delta\tau = 10$, $N = 2$	Initial	0.26894	0.26624
	↑	0.66729	0.62988		↑	1.00000	0.95227
	↓	0.12240	0.16260		↓	0.00000	0.02869
$\delta\tau = 0.5$, $N = 3$	Initial	0.26894	0.24768	$\delta\tau = 10$, $N = 3$	Initial	0.26894	0.26001
	↑	0.69001	0.65955		↑	1.00000	0.95825
	↓	0.11404	0.15076		↓	0.00000	0.03088
	↑↑	0.85104	0.78357		↑↑	1.00000	0.95007
	↑↓	0.33157	0.36841		↑↓	0.00000	0.04944
	↓↑	0.57427	0.51660		↓↑	1.00000	0.93567
	↓↓	0.05480	0.11548		↓↓	0.00000	0.03137
$\delta\tau = 0.5$, $N = 4$	Initial	0.26894	0.26123	$\delta\tau = 10$, $N = 4$	Initial	0.26894	0.25891
	↑	0.70323	0.67090		↑	1.00000	0.94946
	↓	0.10917	0.16821		↓	0.00000	0.03284
	↑↑	0.86494	0.80188		↑↑	1.00000	0.94153
	↑↓	0.32005	0.35596		↑↓	0.00000	0.04114
	↓↑	0.59458	0.51721		↓↑	1.00000	0.95654
	↓↓	0.04969	0.09521		↓↓	0.00000	0.03052
	↑↑↑	0.93362	0.87085		↑↑↑	1.00000	0.93958
	↑↑↓	0.42511	0.36462		↑↑↓	0.00000	0.03894
	↑↓↑	0.66581	0.67371		↑↓↑	1.00000	0.94092
	↑↓↓	0.15730	0.18115		↑↓↓	0.00000	0.03552
	↓↑↑	0.80074	0.75549		↓↑↑	1.00000	0.93787
	↓↑↓	0.29223	0.25769		↓↑↓	0.00000	0.03308
	↓↓↑	0.53293	0.56665		↓↓↑	1.00000	0.93896
	↓↓↓	0.02442	0.06262		↓↓↓	0.00000	0.03613

In the hybrid simulation, we need to implement all the possible choices of the sub-channels. The populations of the system qubit are measured at $t = 0, \delta\tau, \dots, N\delta\tau$ in a N-step isothermal process. With two choices of sub-channel in one step, there are 2^j choices of the sub-channels at the j-th step. Since no work is done in the last step, we only need to obtain the excited state population at $t = 0, \delta\tau, \dots, (N-1)\delta\tau$, where $\delta\tau$ is the time consuming of the isochoric process. We can in principal just implement the $N-1$ steps of elementary processes and the N-th adiabatic process. We do not implement the last isochoric process to reduce the number of the circuits.

We show the circuit implemented on ibmqx2 for different step number $N = 2, 3,$ and 4 with the time consuming $\delta\tau = 0.5$ and $\delta\tau = 10$ in the following. Every circuit is run once with 8192 shots.

In the result, the index of the qubits is organized as 1, 2, 3, 4 and 5. The qubit 3 represents the system qubit, while the rest are the ancillary qubits. The measurement is performed on the system qubit only, with other qubits undetected. In the histograms, the state 0 on qubit (1, 2, 4, 5) is marked for simplicity.

Short-time dynamics with $\delta\tau = 0.5$

We show the ibmqx2 results for the short-time discrete isothermal process with the step number $N = 2, 3,$ and 4.

I. Two-step isothermal process with $\delta\tau = 0.5$

For a two-step isothermal process, we need to obtain the excited state population at $t = 0, \delta\tau$.

1. Prepare the initial state at $t = 0$

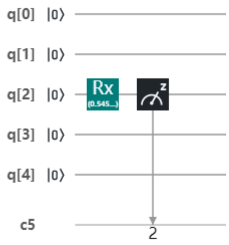

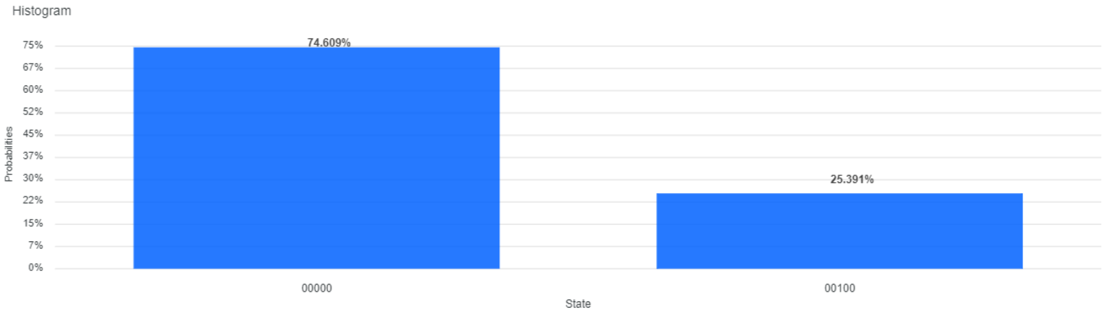

The initial state is prepared with the $R_x(0.54521 \times 2)$ operation. The excited state population is $p_e(0) = 0.25391$. The raw data was obtained on April 16th, 2020.

2. The first step $t = \delta\tau$.

For the sub-channel choice $\uparrow$, the circuit and the result are

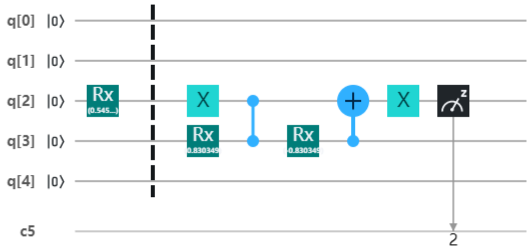

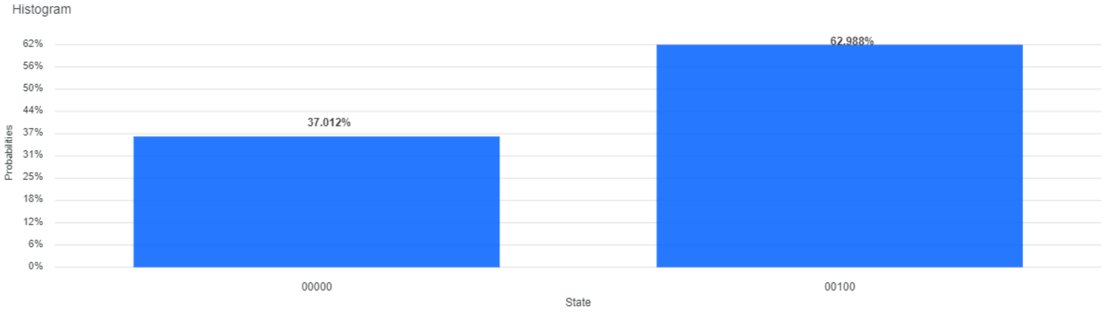

The excited state population is $p_e(\delta\tau) = 0.62988$. The raw data was obtained on April 16th, 2020.

For the sub-channel choice $\downarrow$, the circuit and the result are

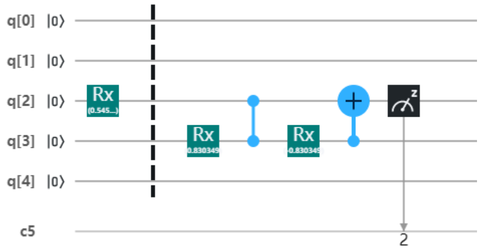

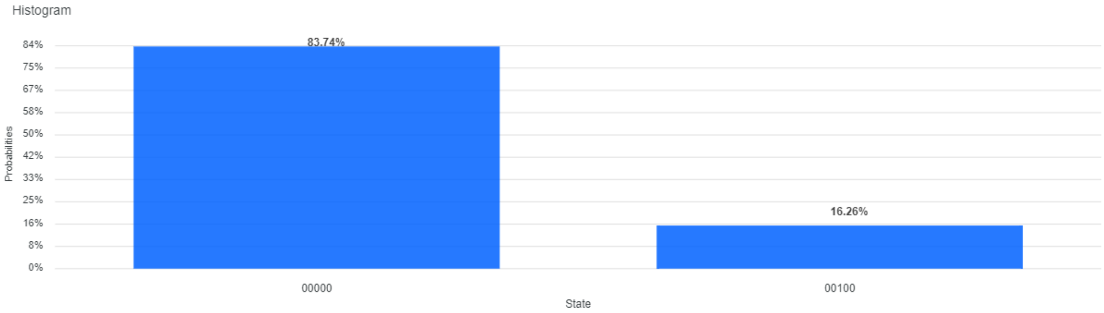

The excited state population is $p_e(\delta\tau) = 0.16260$. The raw data was obtained on April 16th, 2020.

II. Three-step isothermal process with $\delta\tau = 0.5$

For the three-step isothermal process, we need to obtain the excited state population at $t =$

$0, \delta\tau, 2\delta\tau$.

1. Prepare the initial state at $t = 0$

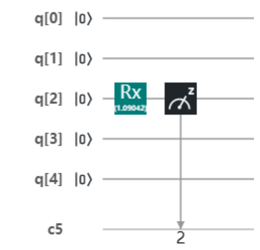

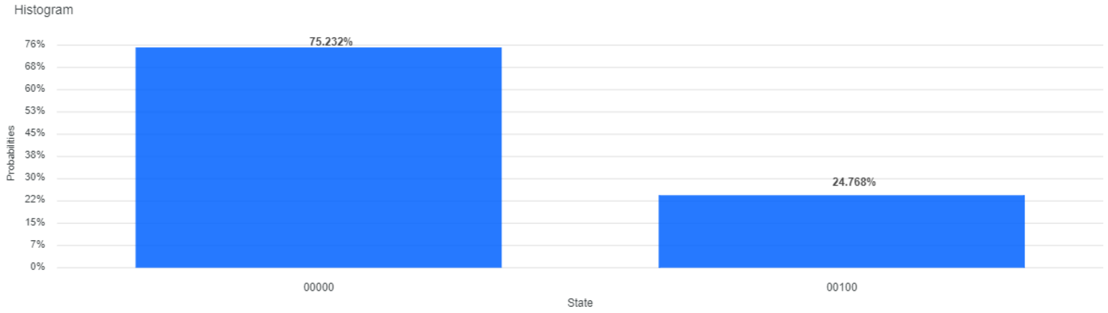

The initial state is prepared with the $R_x(0.54521 \times 2)$ operation. The excited state population is $p_e(0) = 0.24768$. The raw data was obtained on April 16th, 2020.

2. The first step $t = \delta\tau$.

For the sub-channel choice $\uparrow$, the circuit and the result are

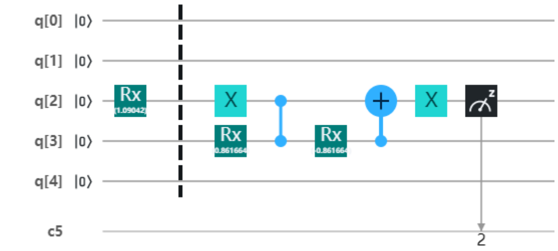

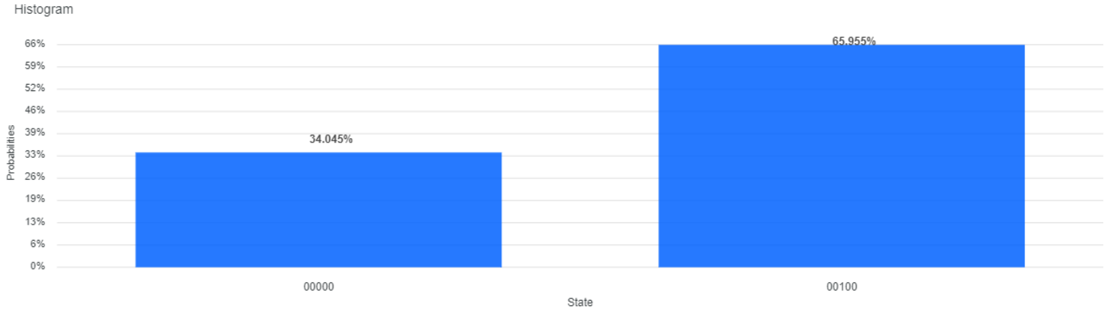

The excited state population is $p_e(\delta\tau) = 0.65955$. The raw data was obtained on April 16th, 2020.

For the sub-channel choice $\downarrow$, the circuit and the results are

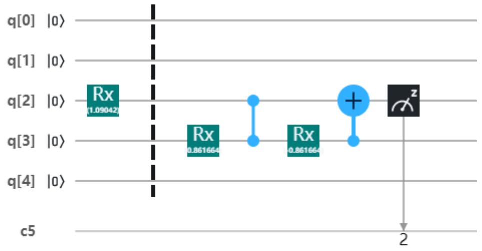

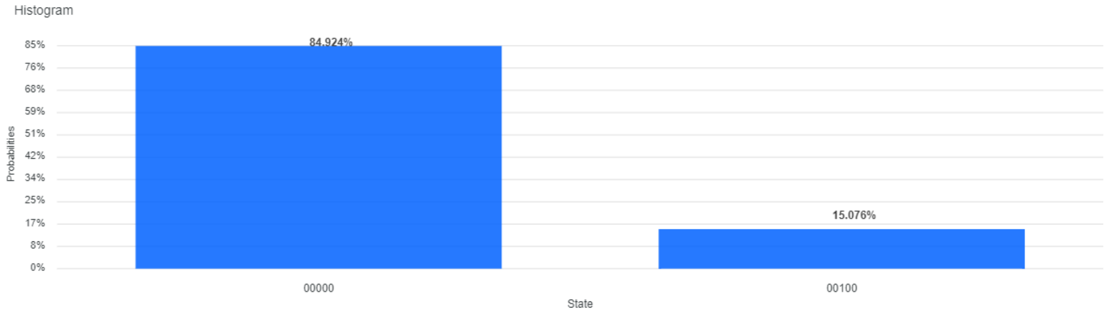

The excited state population is $p_e(\delta\tau) = 0.15076$. The raw data was obtained on April 16th, 2020.

3. The second step $t = 2\delta\tau$.

For the sub-channel choice $\uparrow\uparrow$, the circuit and the result are

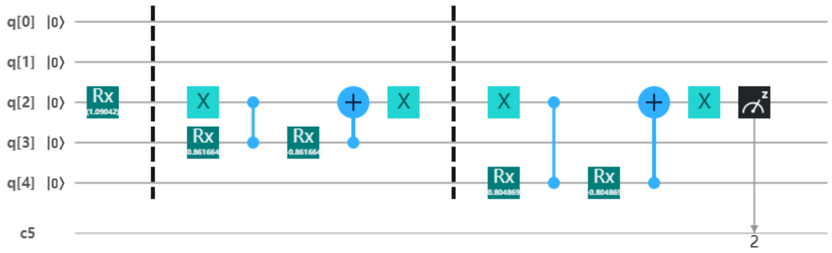

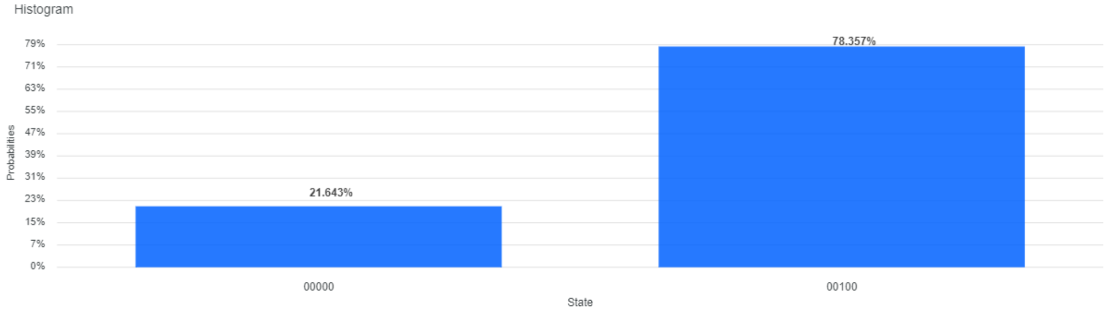

The excited state population $p_e(2\delta\tau) = 0.78357$. The raw data was obtained on April 16th,

2020.

For the sub-channel choice ↑↓, the circuit and the result are

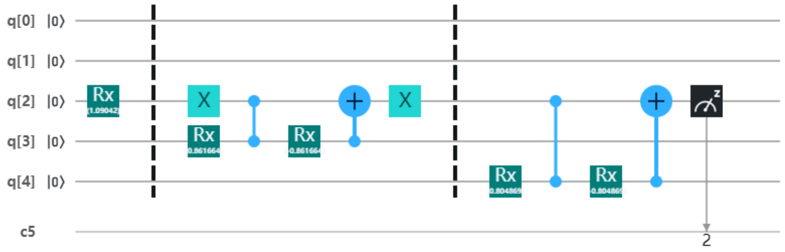

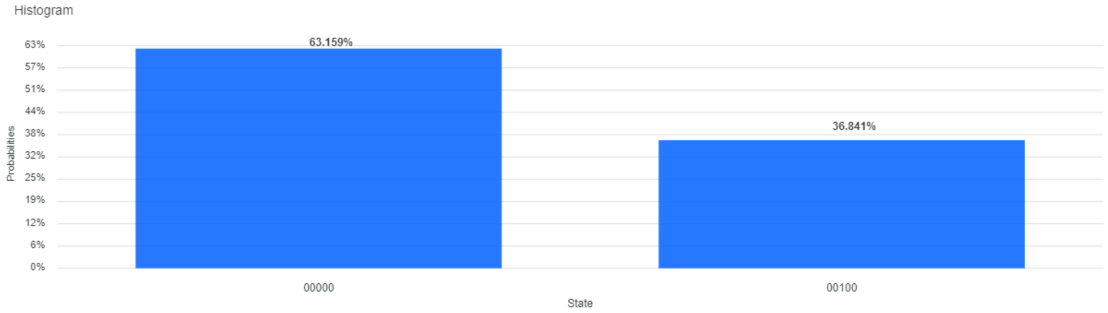

The excited state population is $p_e(2\delta\tau) = 0.36841$. The raw data was obtained on April 16th, 2020.

For the sub-channel choice ↓↑, the circuit and the result are

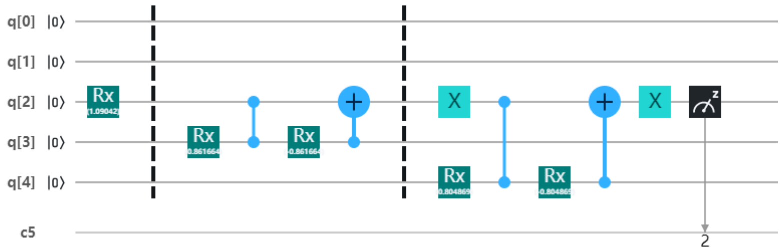

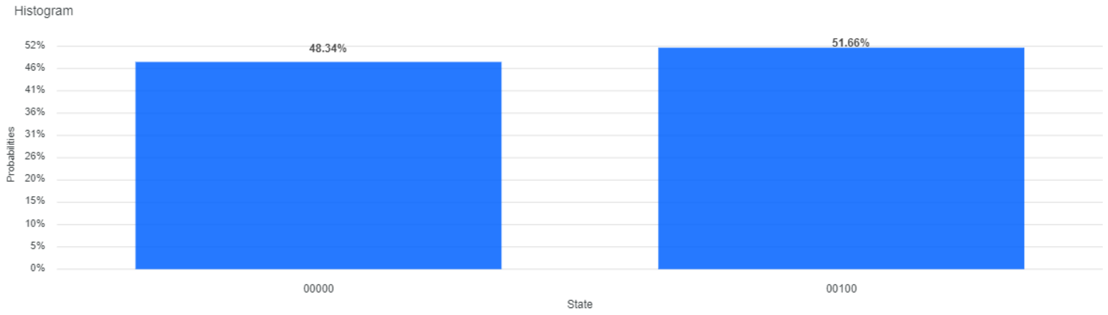

The excited state population is $p_e(2\delta\tau) = 0.51660$. The raw data was obtained on April 17th, 2020.

For the sub-channel choice ↓↓, the circuit and the result are

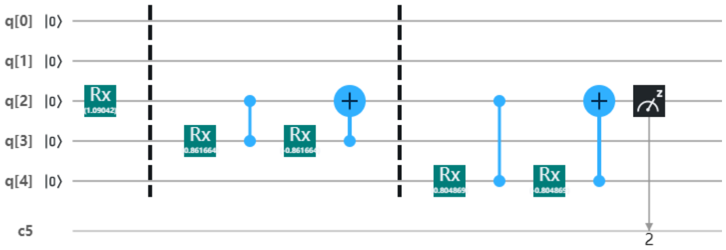

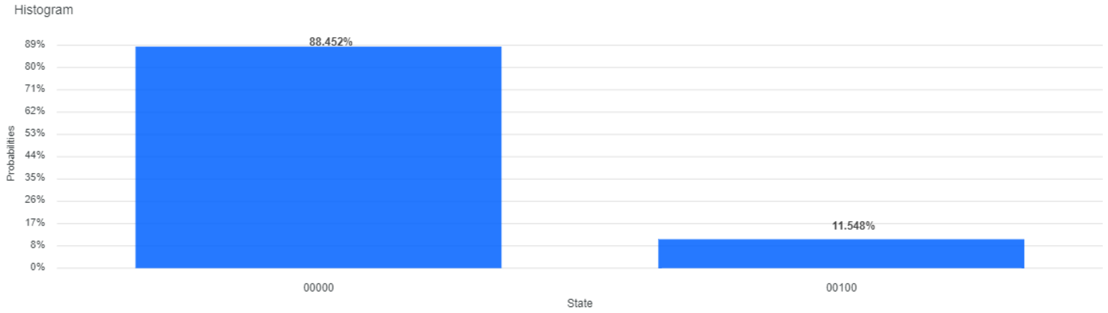

The excited state population is $p_e(2\delta\tau) = 0.11548$. The raw data was obtained on April 17th, 2020.

III. Four-step isothermal process with $\delta\tau = 0.5$

For the four-step isothermal process, we need to obtain the excited state population at $t = 0, \delta\tau, 2\delta\tau, 3\delta\tau$.

1. Prepare the initial state at $t = 0$.

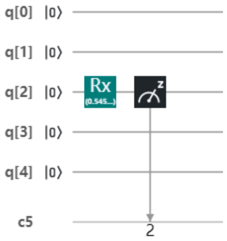

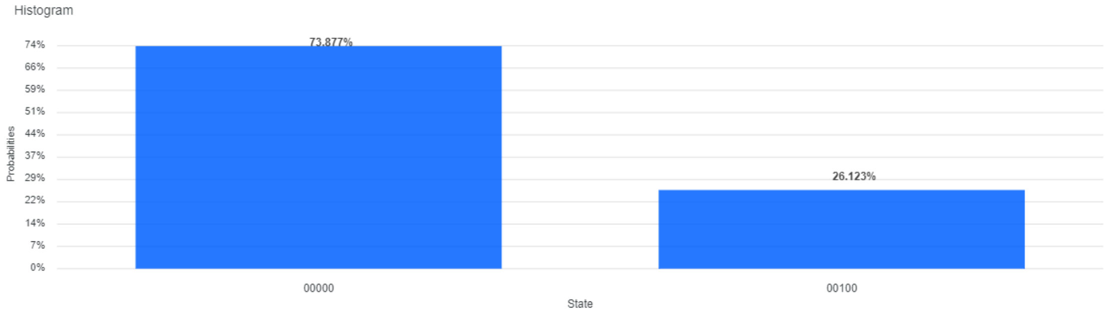

The initial state is prepared with the $R_x(0.54521 \times 2)$ operation. The excited state population

is $p_e(0) = 0.26123$. The raw data was obtained on April 17th, 2020.

2. The first step $t = \delta\tau$.

For the sub-channel choice $\uparrow$, the circuit and the result are

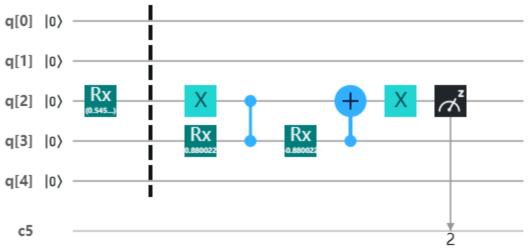

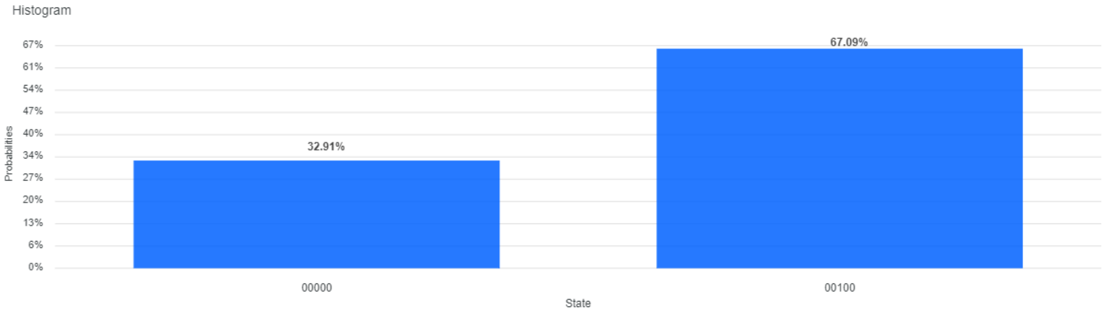

The excited state population is $p_e(\delta\tau) = 0.67090$. The raw data was obtained on April 17th, 2020.

For the sub-channel choice $\downarrow$, the circuit and the result are

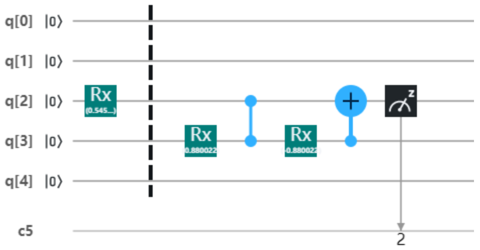

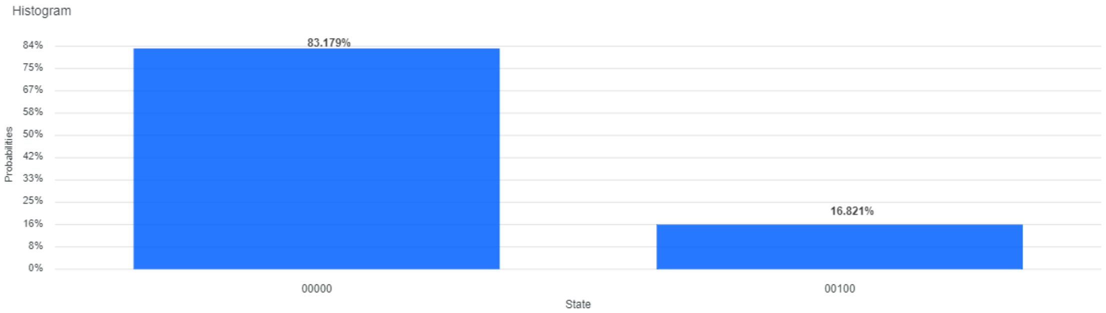

The excited state population $p_e(\delta\tau) = 0.16821$. The raw data was obtained on April 17th, 2020.

3. The second step $t = 2\delta\tau$.

For the sub-channel choice ↑↑, the circuit and the result are

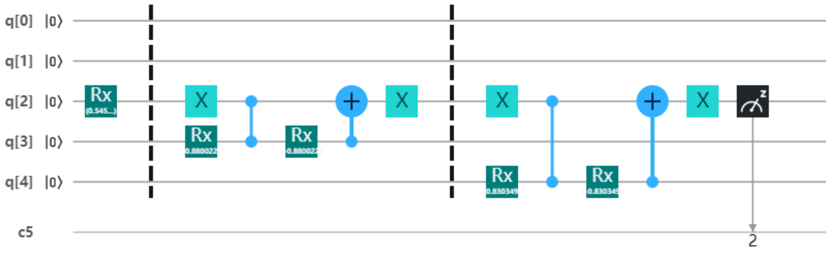

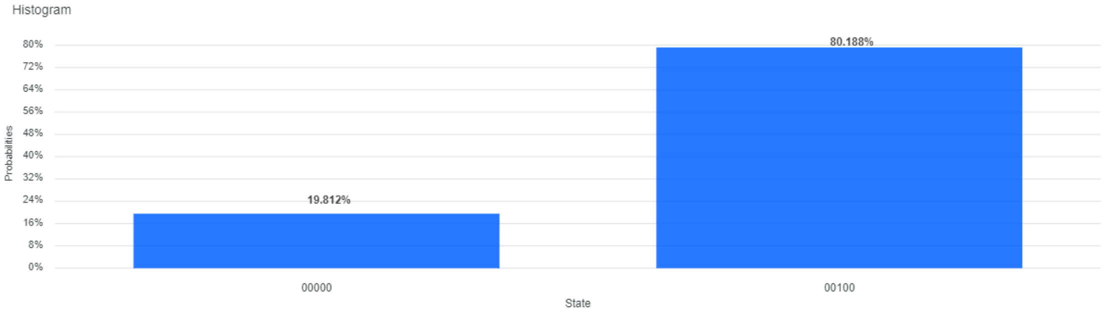

The excited state population is $p_e(2\delta\tau) = 0.80188$. The raw data was obtained on April 17th, 2020.

For the sub-channel choice ↑↓, the circuit and the result are

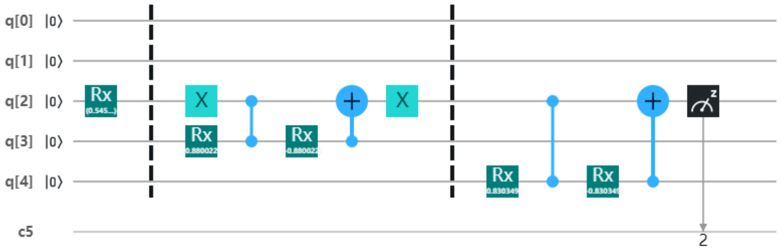

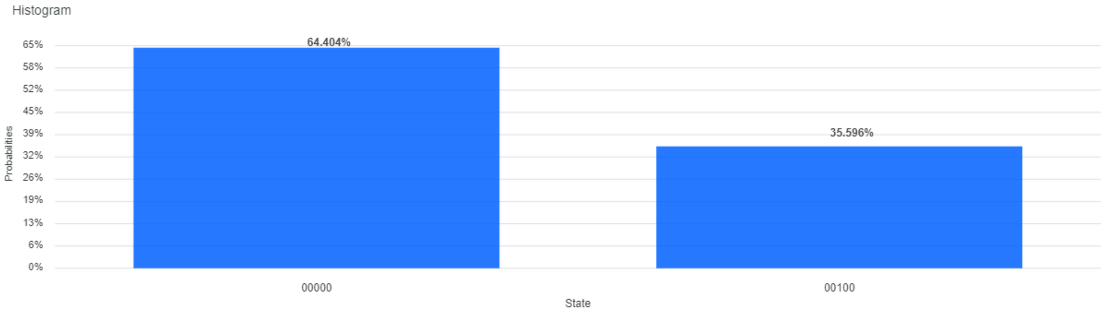

The excited state population is $p_e(2\delta\tau) = 0.35596$. The raw data was obtained on April 17th, 2020.

For the sub-channel choice ↓↑, the circuit and the result are

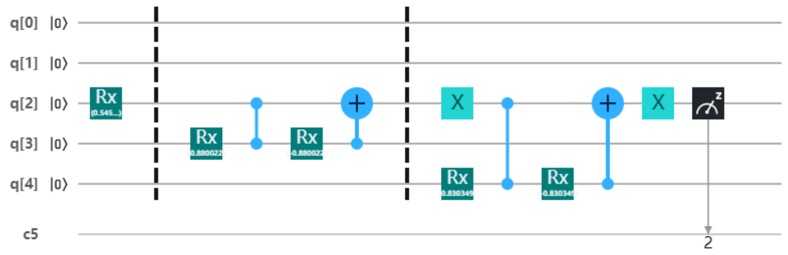

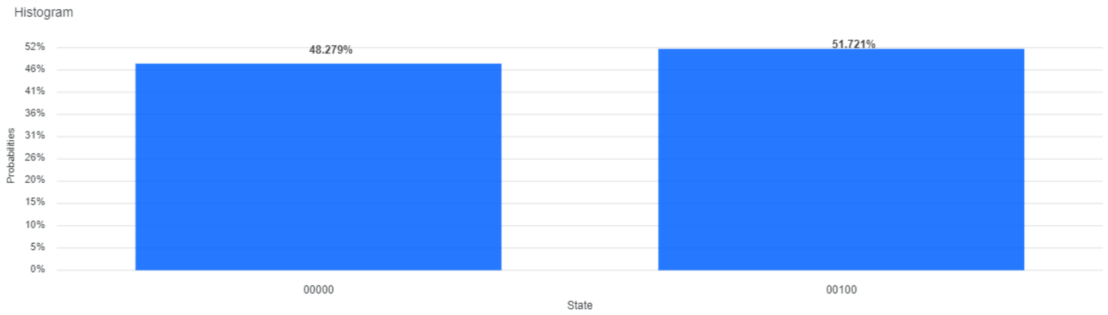

The excited state population is $p_e(2\delta\tau) = 0.51721$. The raw data was obtained on April 17th, 2020.

For the sub-channel choice ↓↓, the circuit and the result are

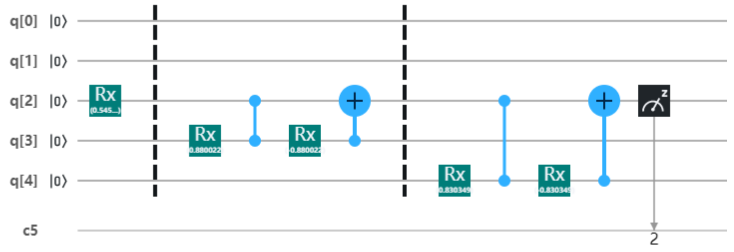

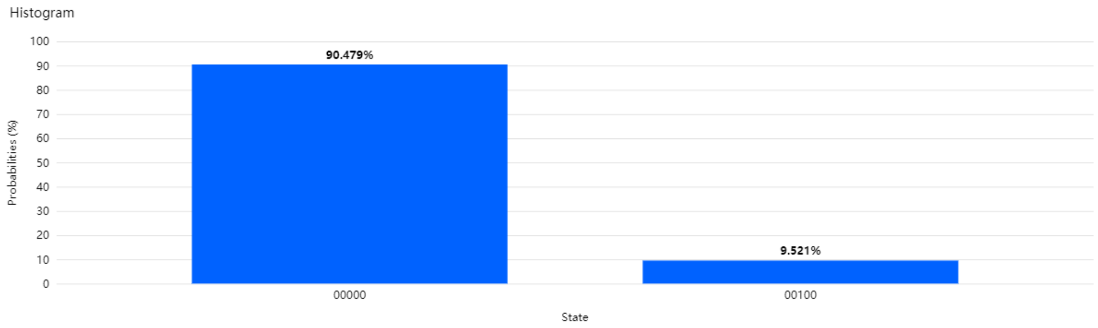

The excited state population is $p_e(2\delta\tau) = 0.09521$. The raw data was obtained on April 17th, 2020.

4. The third step $t = 3\delta\tau$.

For the sub-channel choice ↑↑↑, the circuit and the result are

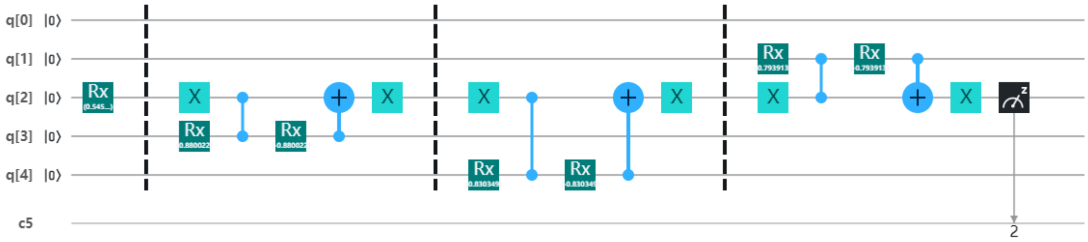

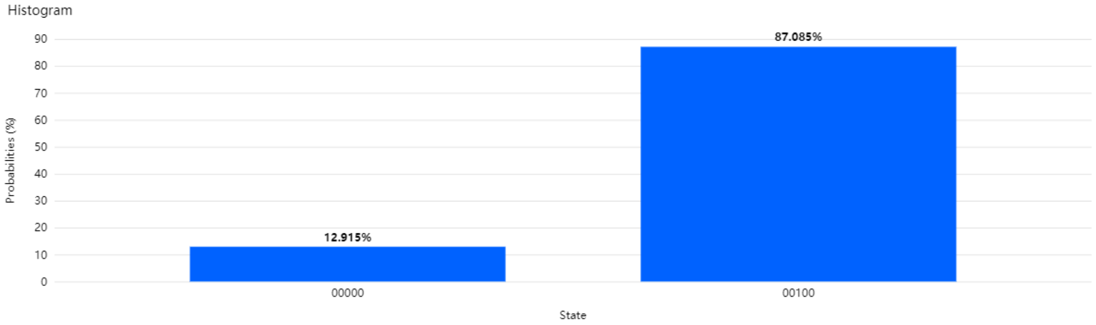

The excited state population is $p_e(3\delta\tau) = 0.87085$. The raw data was obtained on April 17th, 2020.

For the sub-channel choice ↑↑↓, the circuit and the result are

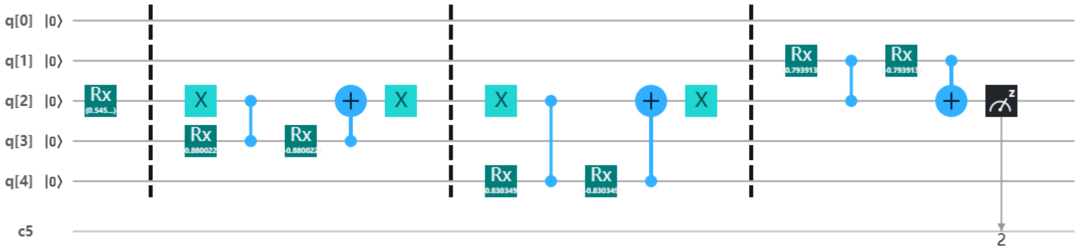

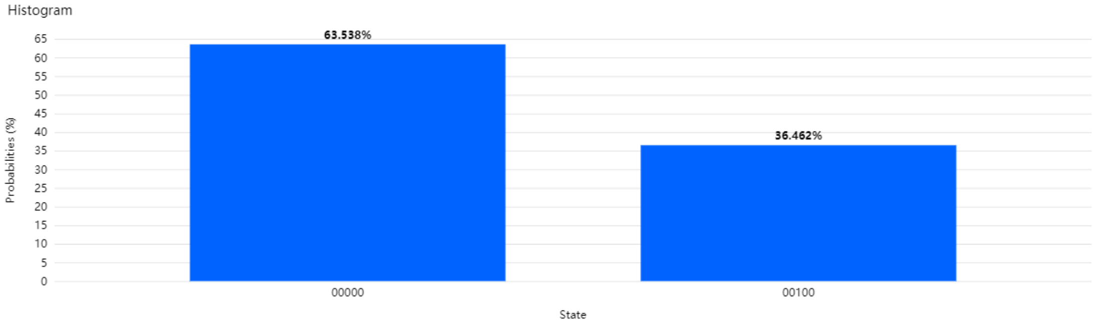

The excited state population is $p_e(3\delta\tau) = 0.36462$. The raw data was obtained on April 17th, 2020.

For the sub-channel choice ↑↓↑, the circuit and the result are

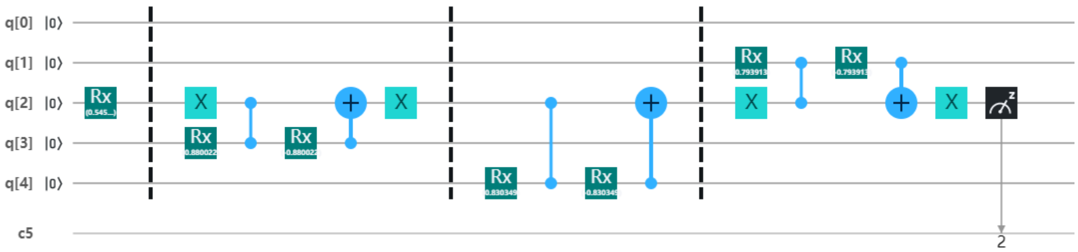

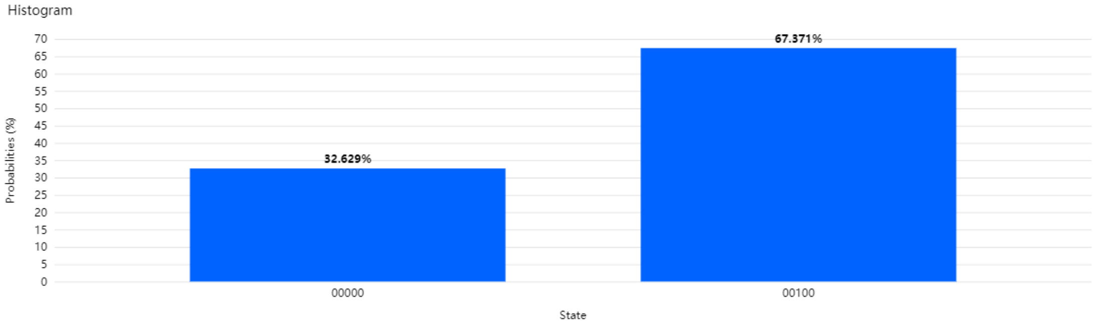

The excited state population is $p_e(3\delta\tau) = 0.67371$. The raw data was obtained on April 17th, 2020.

For the sub-channel choice ↑↓↓, the circuit and the result are

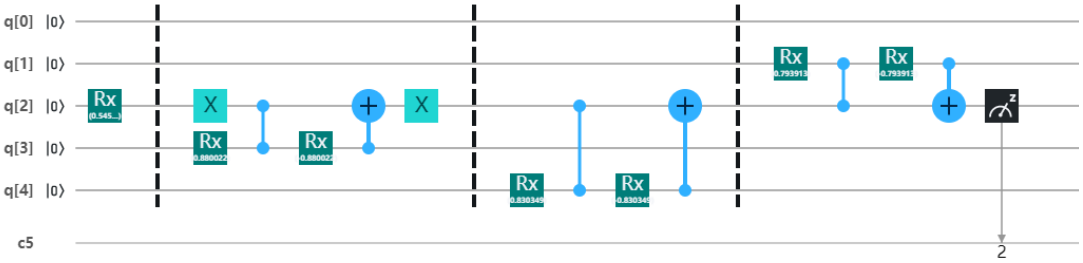

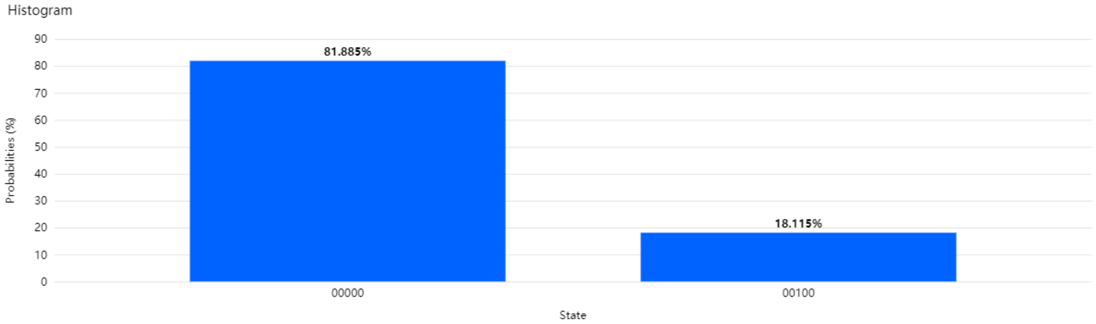

The excited state population is $p_e(3\delta\tau) = 0.18115$. The raw data was obtained on April 17th, 2020.

For the sub-channel choice ↓↑↑, the circuit and the result are

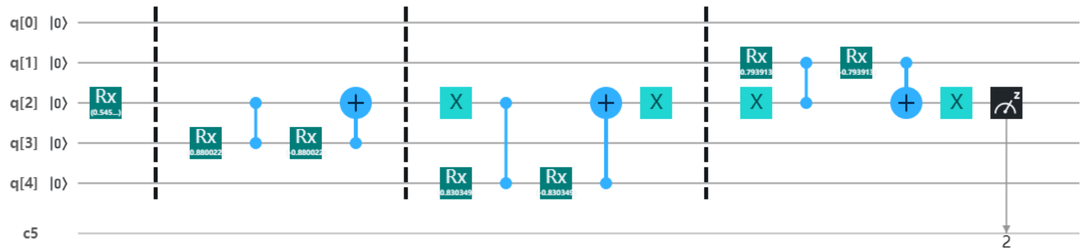

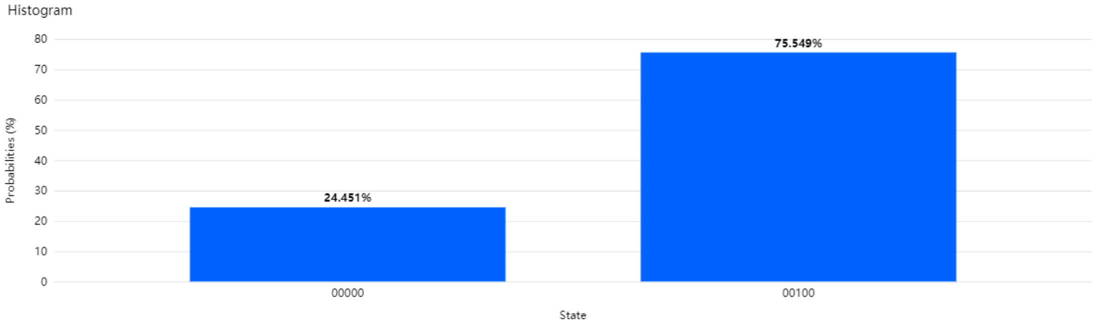

The excited state population is $p_e(3\delta\tau) = 0.75549$. The raw data was obtained on April 17th, 2020.

For the sub-channel choice ↓↑↓, the circuit and the result are

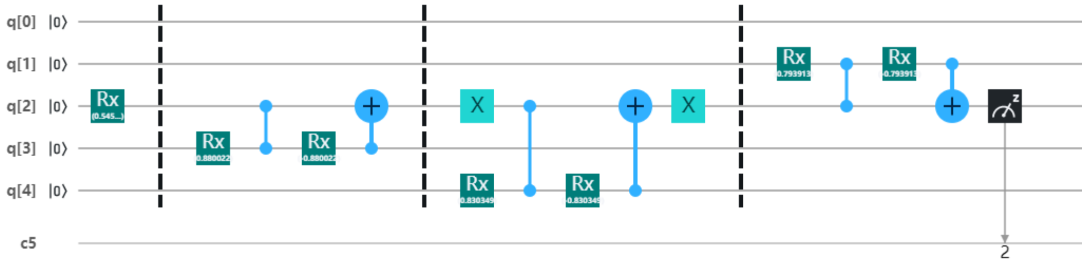

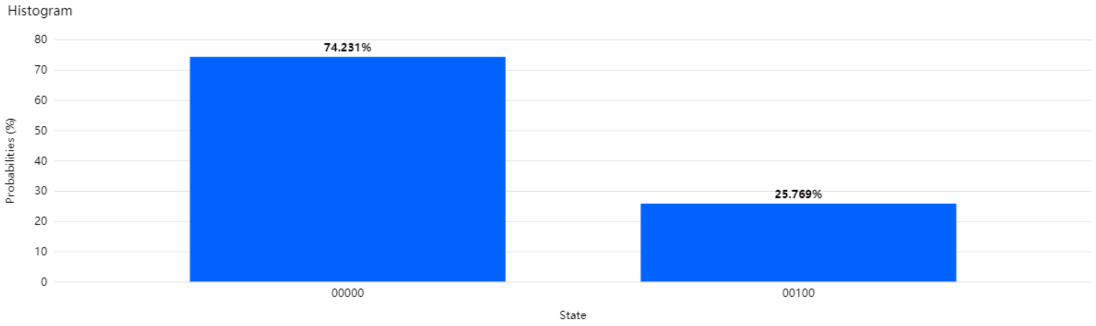

The excited state population is $p_e(3\delta\tau) = 0.25769$. The raw data was obtained on April 17th, 2020.

For the sub-channel choice ↓↓↑, the circuit and the result are

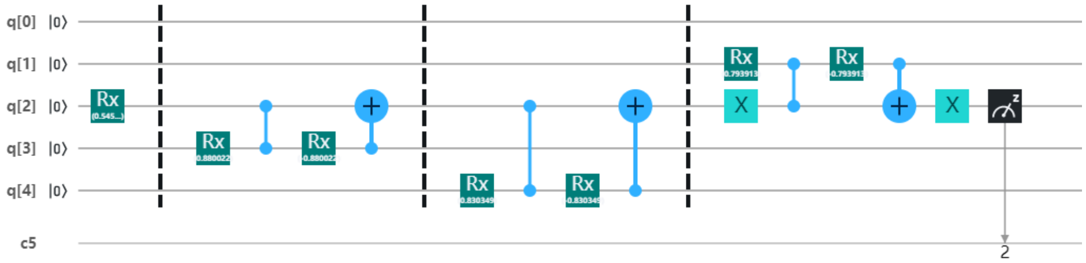

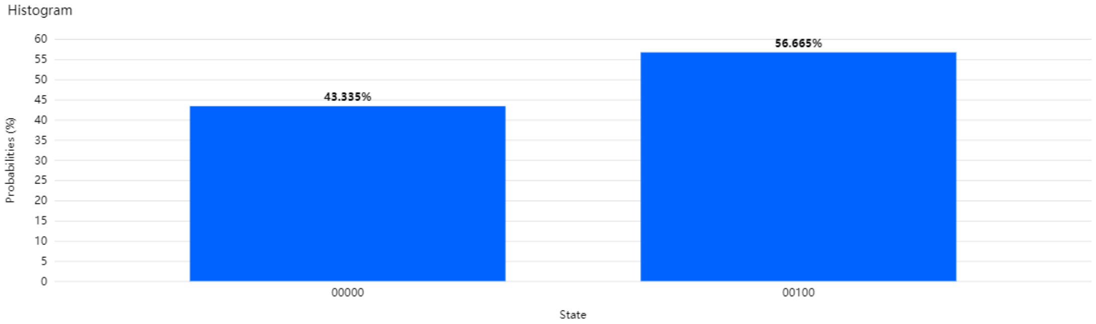

The excited state population is $p_e(3\delta\tau) = 0.56665$. The raw data was obtained on April 17th, 2020.

For the sub-channel choice ↓↓↓, the circuit and the result are

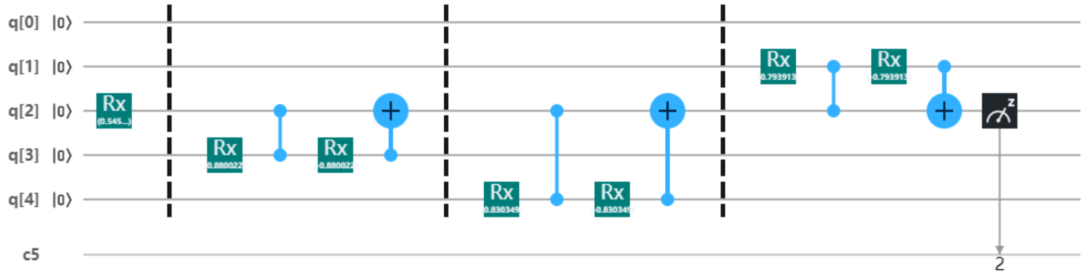

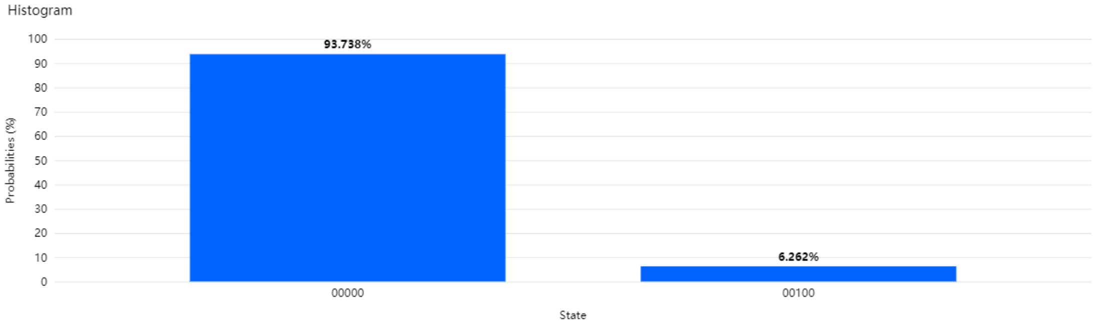

The excited state population is $p_e(3\delta\tau) = 0.06262$. The raw data was obtained on April 17th, 2020.

Long-time dynamics with $\delta\tau = 10$

We next show the ibmqx2 results for the long-time discrete isothermal process with the step number $N = 2,\ 3,$ and 4.

I. Two-step isothermal process $\delta\tau = 10$

1. Prepare the initial state at $t = 0$.

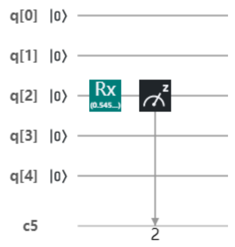

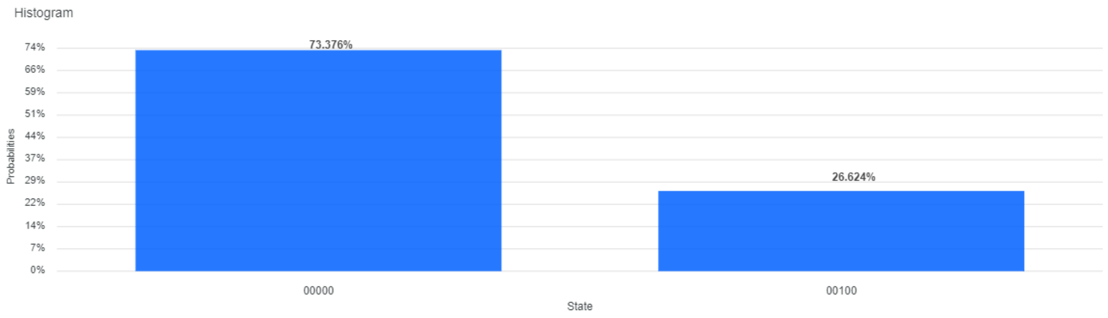

The initial state is prepared with the $R_x(0.54521 \times 2)$ operation. The excited state population is $p_e(0) = 0.26624$. The raw data was obtained on April 17th, 2020.

2. The first step $t = \delta\tau$.

For the sub-channel choice ↑, the circuit and the result are

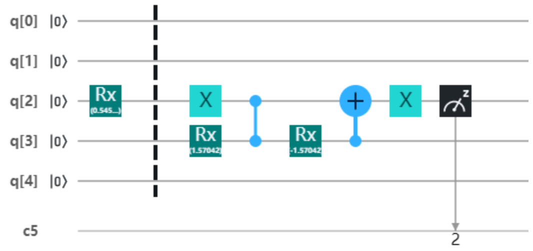

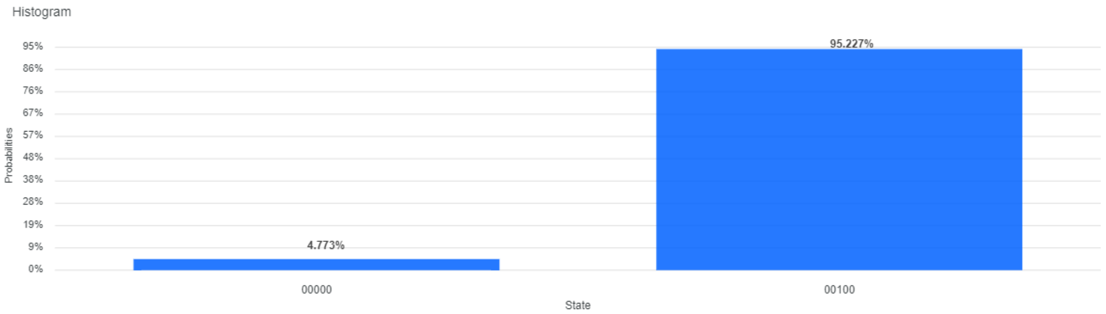

The excited state population is $p_e(\delta\tau) = 0.95227,$ the raw data was obtained on April 17th, 2020.

For the sub-channel choice ↓, the circuit and the result are

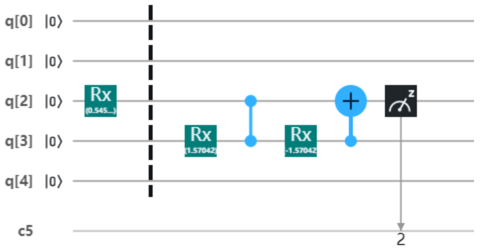

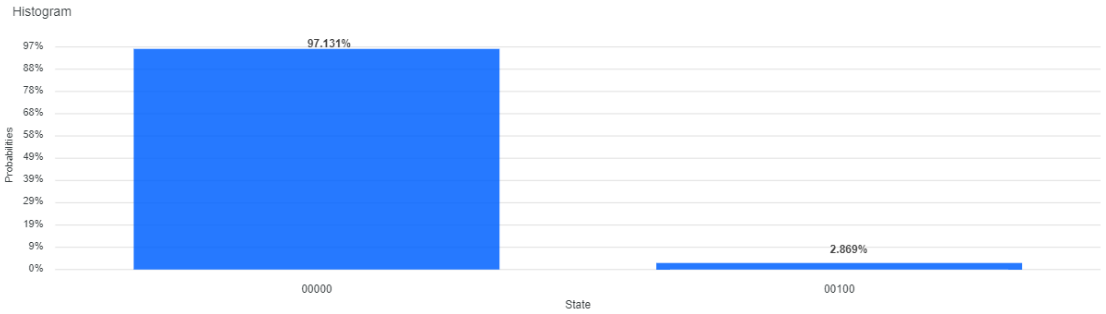

The excited state population is $p_e(\delta\tau) = 0.02869,$ the raw data was obtained on April 17th, 2020.

II. Three-step isothermal process $\delta\tau = 10$

1. Prepare the initial state at $t = 0$.

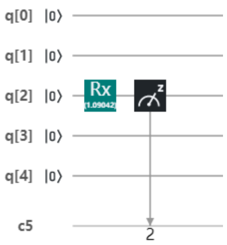

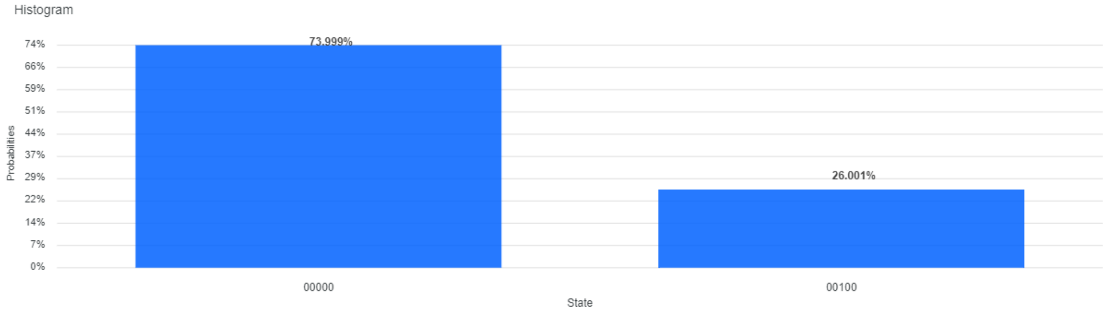

The initial state is prepared with the $R_x(1.09042)$ operation. The excited state population is $p_e(0) = 0.26001$. The raw data was obtained on April 17th, 2020.

2. The first step $t = \delta\tau$.

For the sub-channel choice $\uparrow$, the circuit and the result are

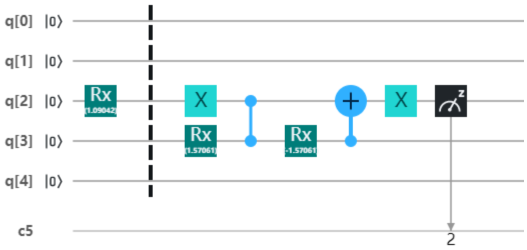

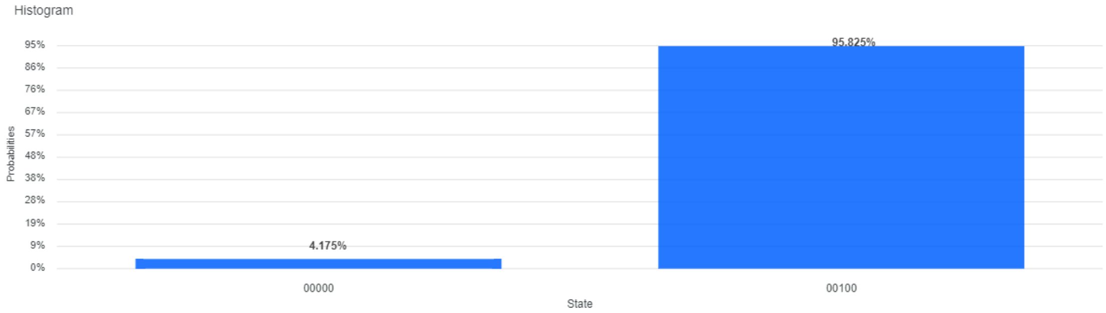

The excited state population is $p_e(\delta\tau) = 0.95825$, the raw data was obtained on April 17th, 2020.

For the sub-channel choice ↓, the circuit and the result are

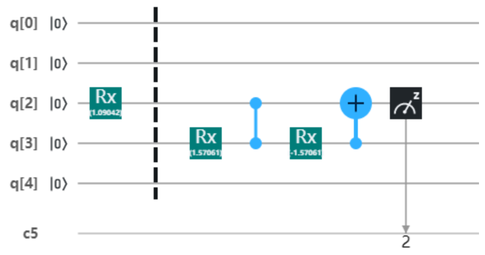

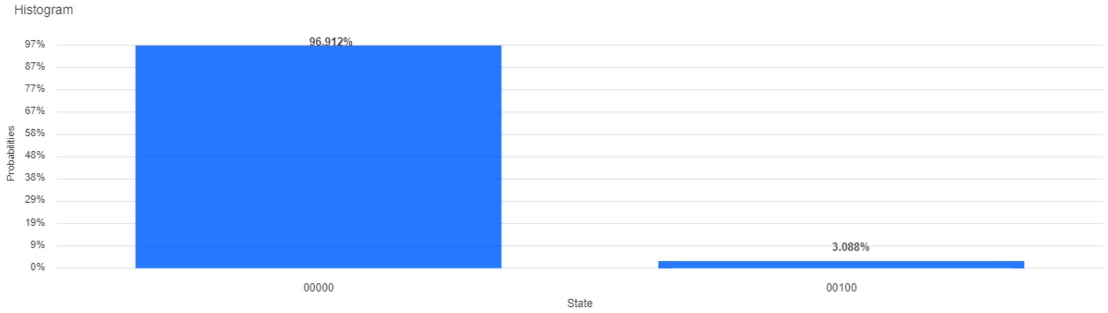

The excited state population is $p_e(\delta\tau) = 0.03088,$ the raw data was obtained on April 17th, 2020.

3. The second step $t = 2\delta\tau$.

For the sub-channel choice ↑↑, the circuit and the result are

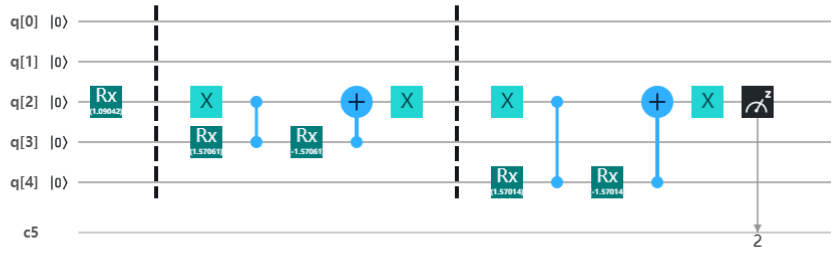

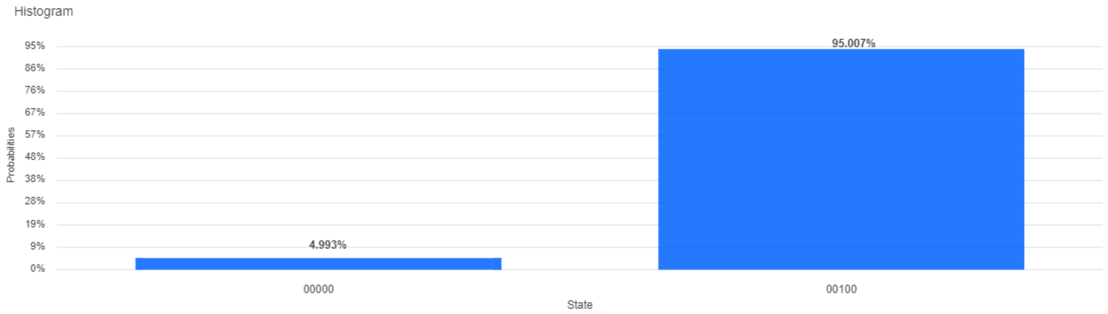

The excited state population is $p_e(2\delta\tau) = 0.95007,$ the raw data was obtained on April 17th, 2020.

For the sub-channel choice ↑↓, the circuit and the result are

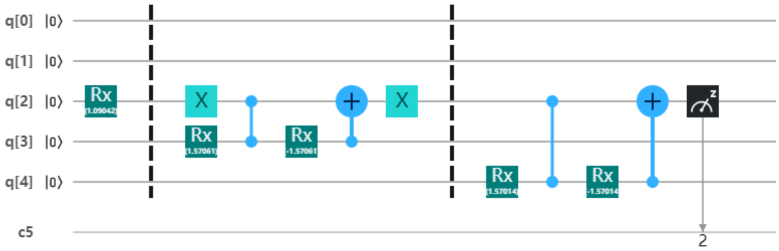

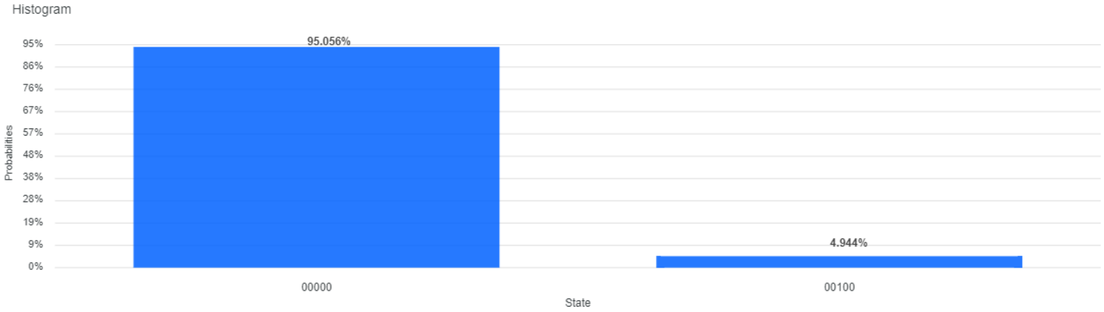

The excited state population is $p_e(2\delta\tau) = 0.04944$, the raw data was obtained on April 17th, 2020.

For the sub-channel choice $\downarrow\uparrow$, the circuit and the result are

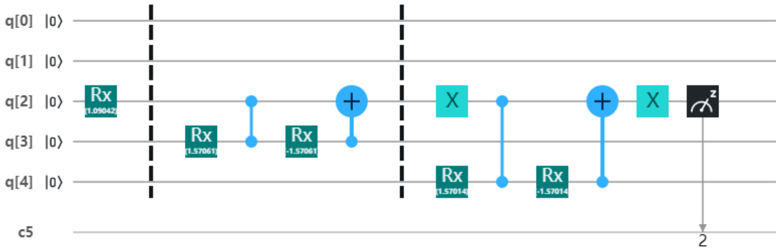

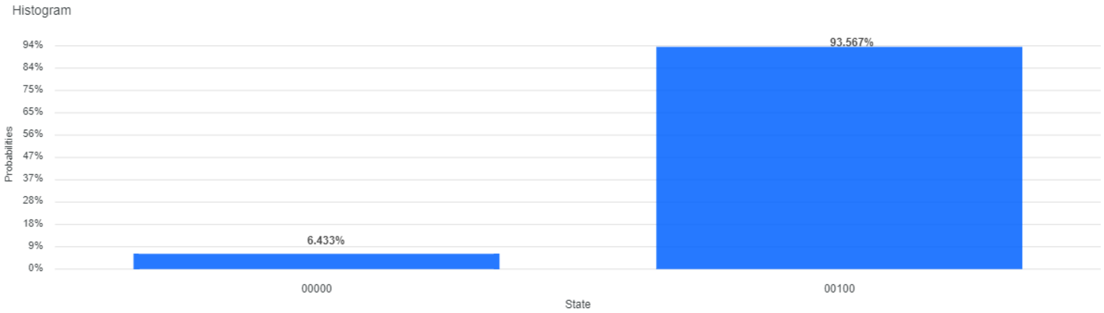

The excited state population is $p_e(2\delta\tau) = 0.93567$, the raw data was obtained on April 17th, 2020.

For the sub-channel choice $\downarrow\downarrow$, the circuit and the result are

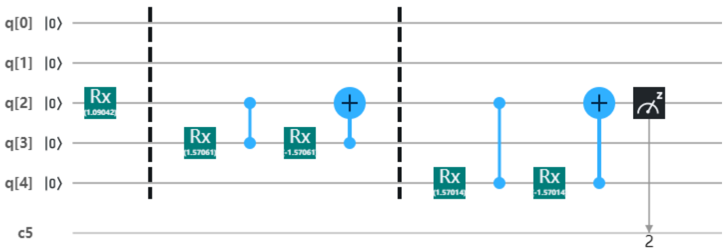

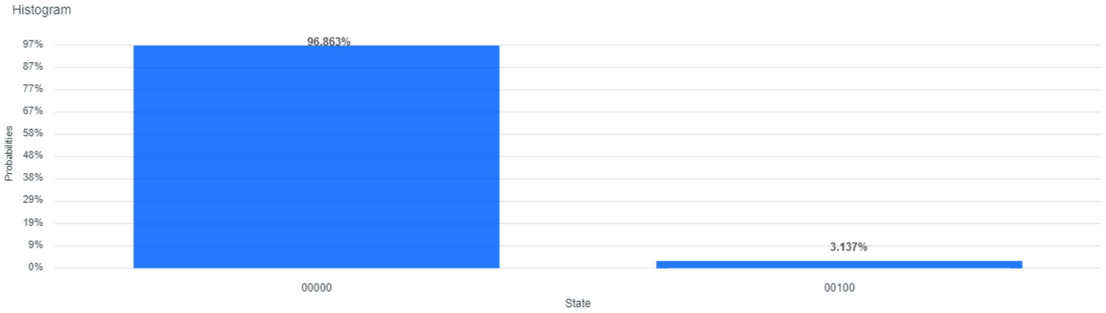

The excited state population is $p_e(2\delta\tau) = 0.03137,$ the raw data was obtained on April 17th, 2020.

III. Four-step isothermal process $\delta\tau = 10$

1. Prepare the initial state at $t = 0$.

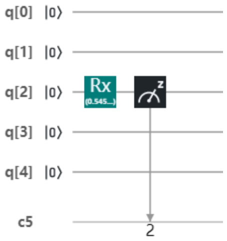

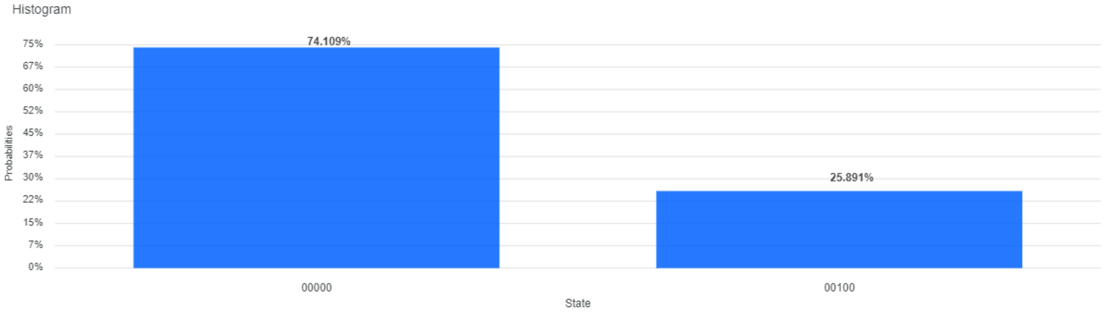

The initial state is prepared with the $R_x(0.54521 \times 2)$ operation. The excited state population is $p_e(0) = 0.25891$. The raw data was obtained on April 17th, 2020.

2. The first step $t = \delta\tau$.

For the sub-channel choice $\uparrow$, the circuit and the result are

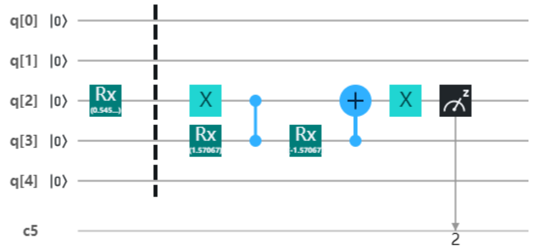

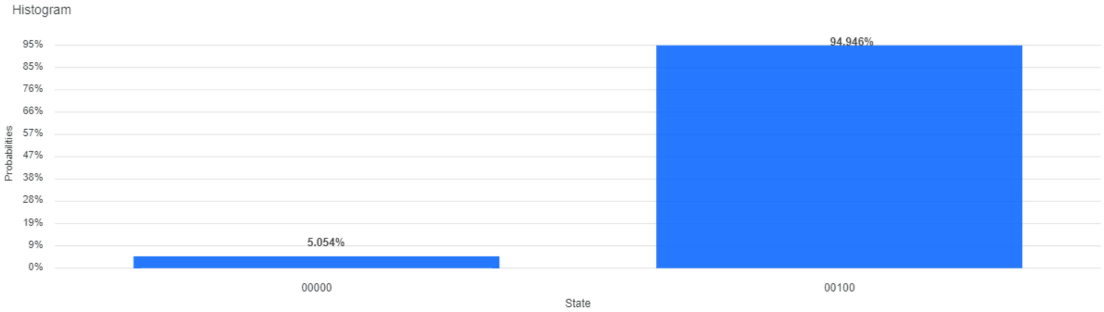

The excited state population is $p_e(\delta\tau) = 0.94946,$ the raw data was obtained on April 17th, 2020.

For the sub-channel choice $\downarrow$, the circuit and the result are

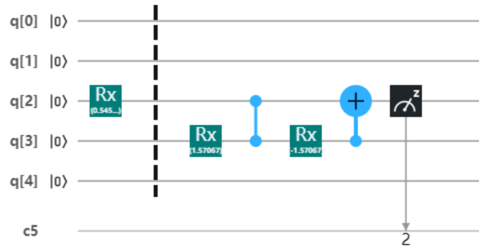

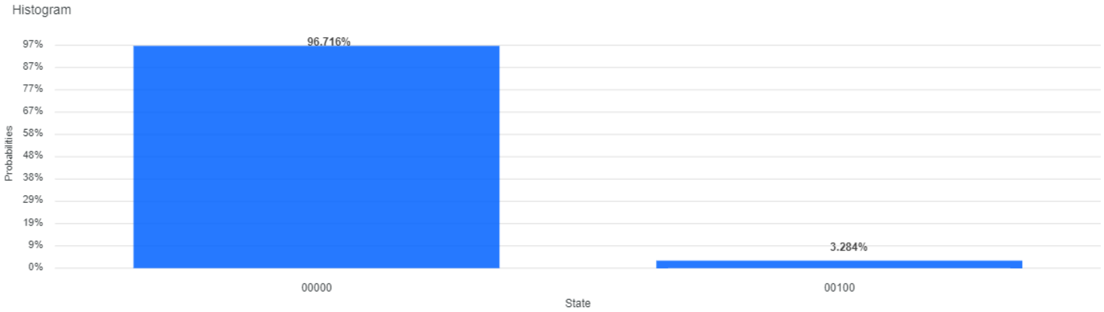

The excited state population is $p_e(\delta\tau) = 0.03284,$ the raw data was obtained on April 17th, 2020.

3. The second step $t = 2\delta\tau$.

For the sub-channel choice $\uparrow\uparrow$, the circuit and the result are

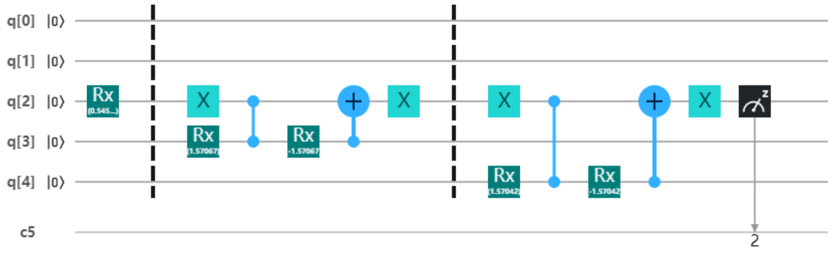

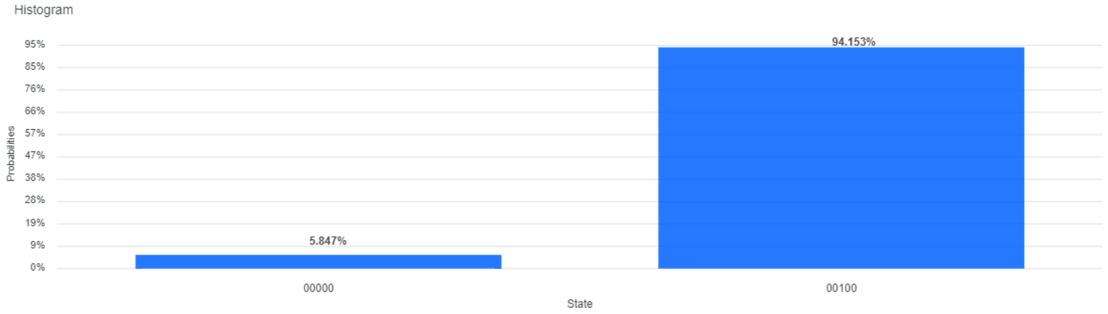

The excited state population is $p_e(2\delta\tau) = 0.94153$, the raw data was obtained on April 17th, 2020.

For the sub-channel choice $\uparrow\downarrow$, the circuit and the result are

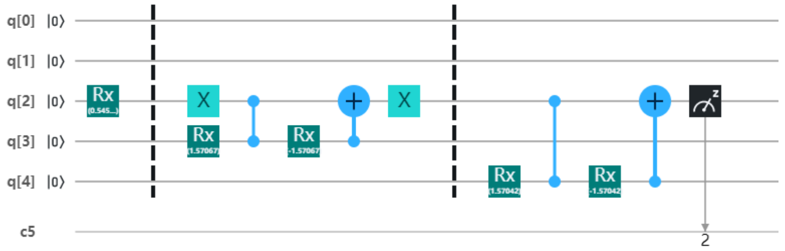

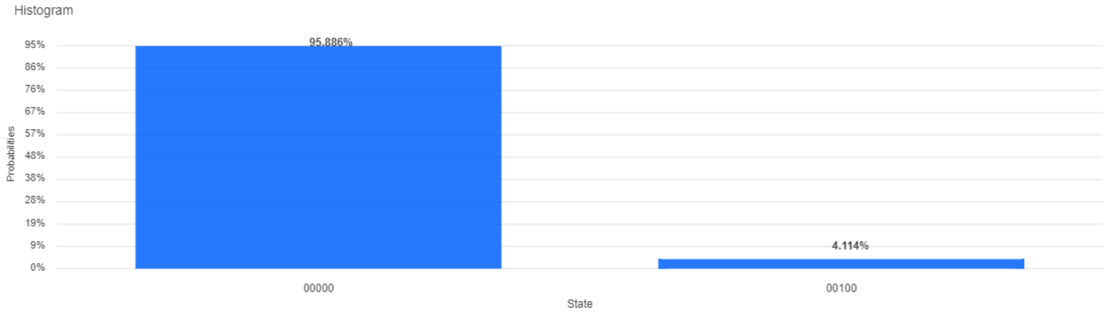

The excited state population is $p_e(2\delta\tau) = 0.04114$, the raw data was obtained on April 17th, 2020.

For the sub-channel choice $\downarrow\uparrow$, the circuit and the result are

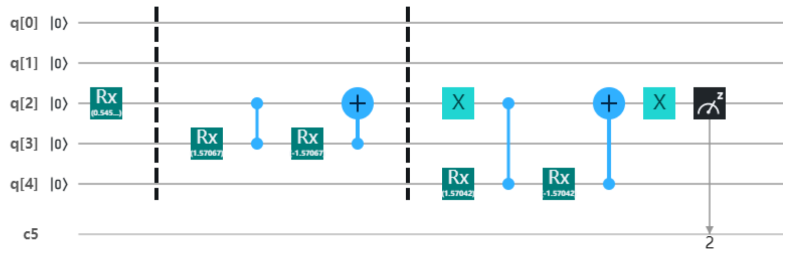

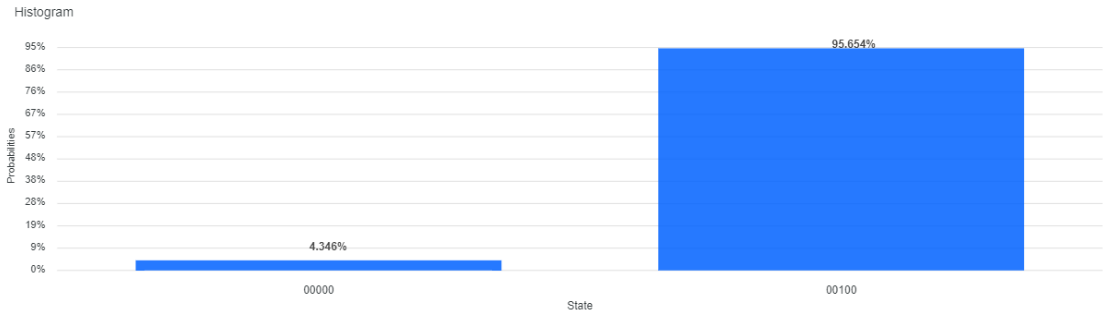

The excited state population is $p_e(2\delta\tau) = 0.95654$, the raw data was obtained on April 17th, 2020.

For the sub-channel choice ↓↓, the circuit and the result are

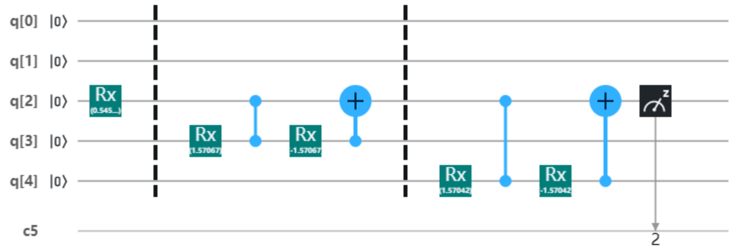

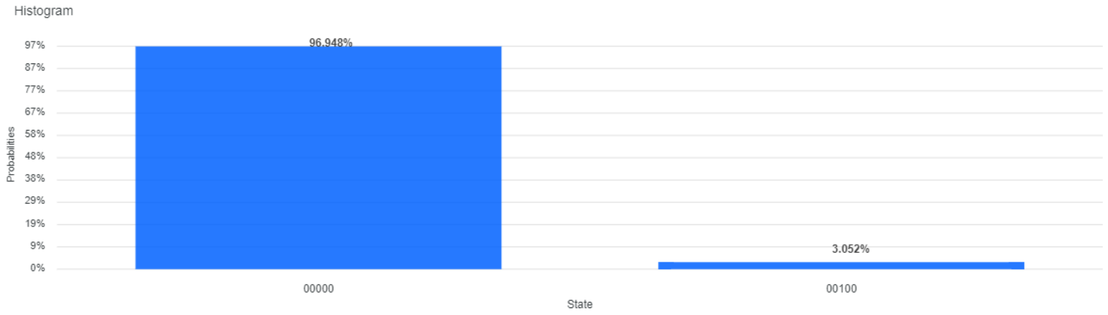

The excited state population is $p_e(2\delta\tau) = 0.03052$, the raw data was obtained on April 17th, 2020.

4. The third step $t = 3\delta\tau$.

For the sub-channel choice ↑↑↑, the circuit and the result are

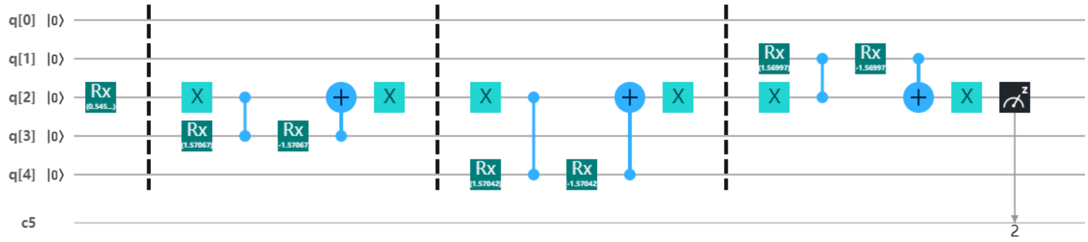

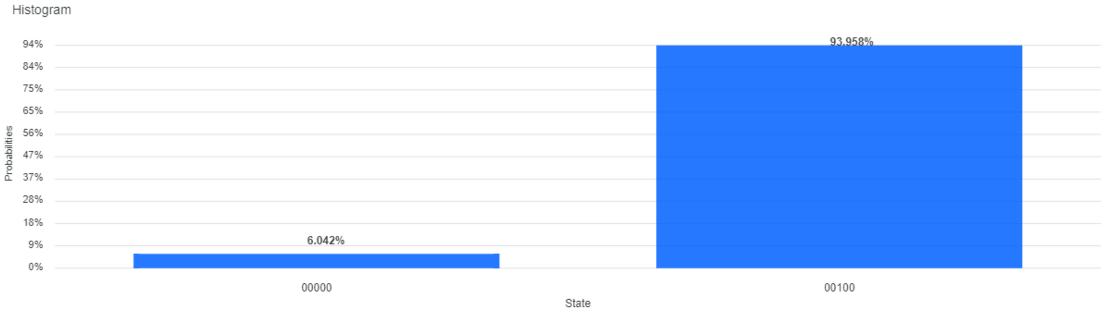

The excited state population is $p_e(3\delta\tau) = 0.93958$, the raw data was obtained on April 17th, 2020.

For the sub-channel choice ↑↑↓, the circuit and the result are

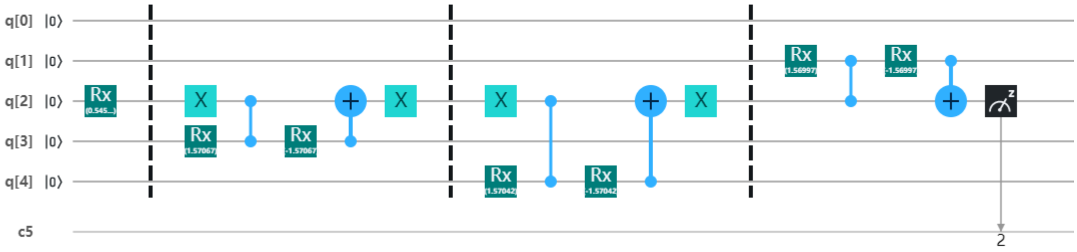

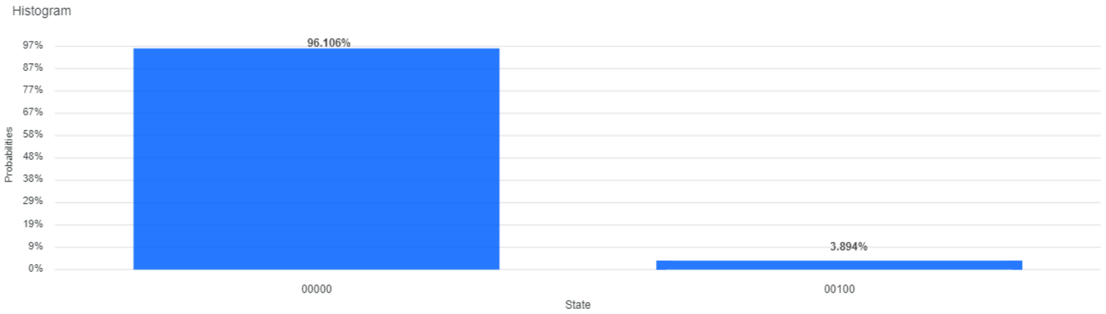

The excited state population is $p_e(3\delta\tau) = 0.03894$. The raw data was obtained on April 17th, 2020.

For the sub-channel choice ↑↓↑, the circuit and the result are

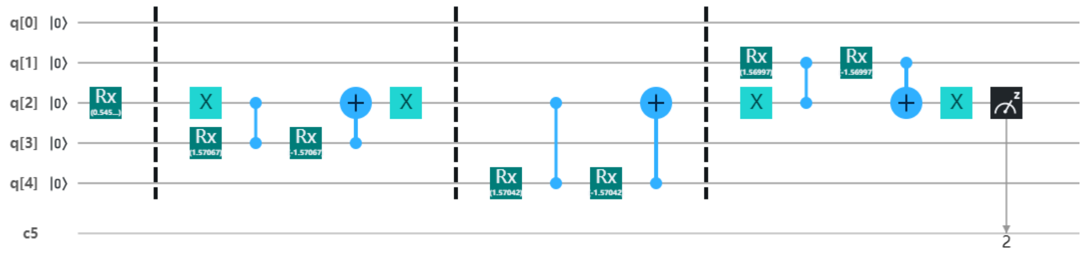

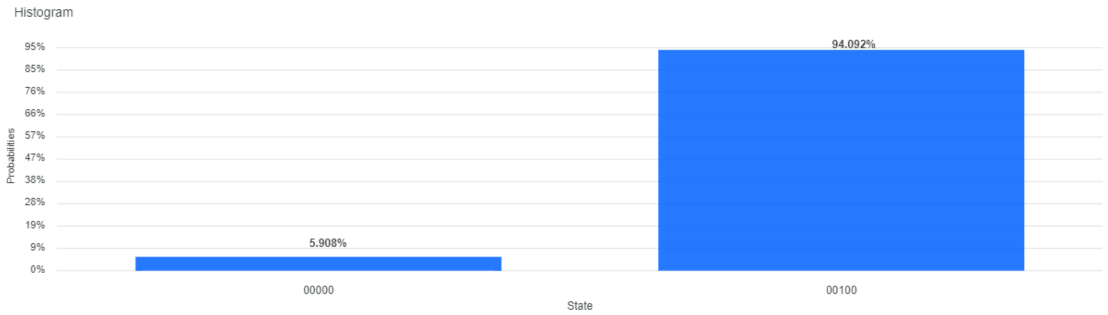

The excited state population $p_e(3\delta\tau) = 0.94092$, the raw data was obtained on April 17th, 2020.

For the sub-channel choice ↑↓↓, the circuit and the result are

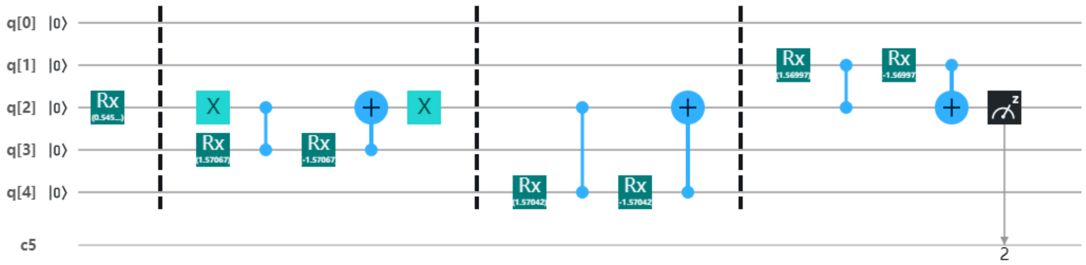

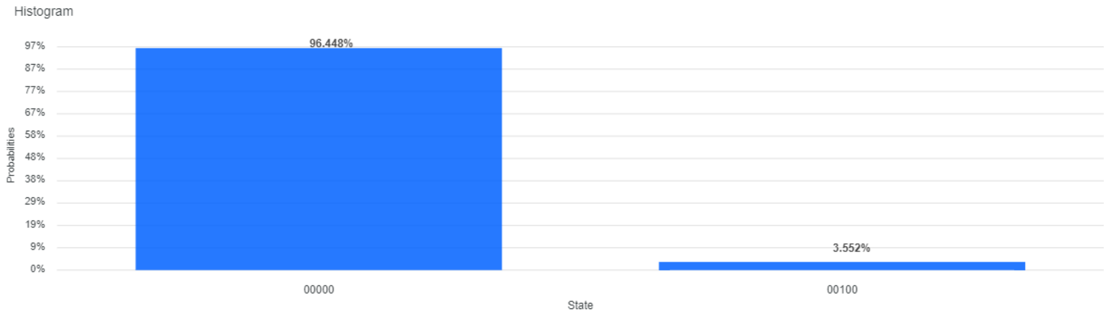

The excited state population is $p_e(3\delta\tau) = 0.03552$. The raw data was obtained on April 17th, 2020.

For the sub-channel choice ↓↑↑, the circuit and the result are

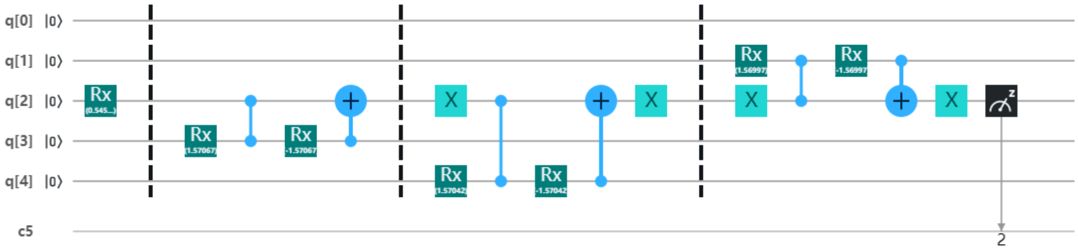

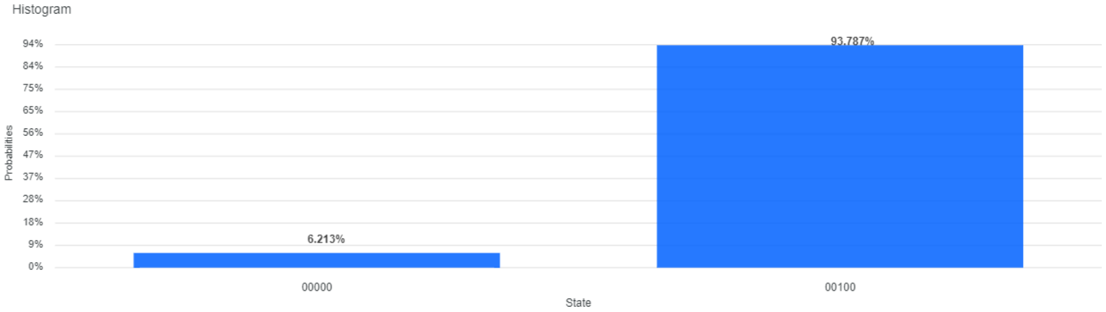

The excited state population is $p_e(3\delta\tau) = 0.93787$. The raw data was obtained on April 17th, 2020.

For the sub-channel choice ↓↑↓, the circuit and the result are

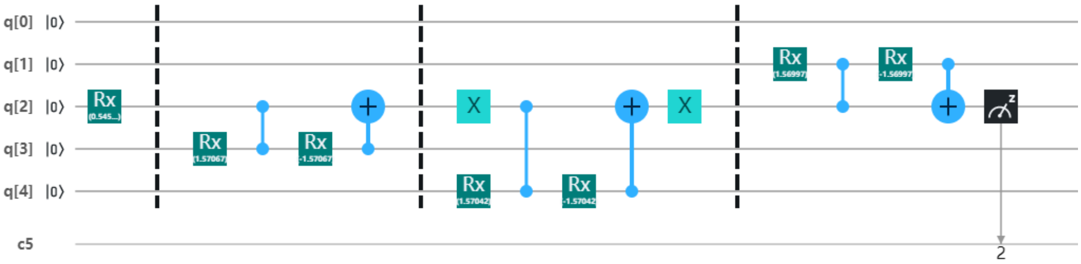

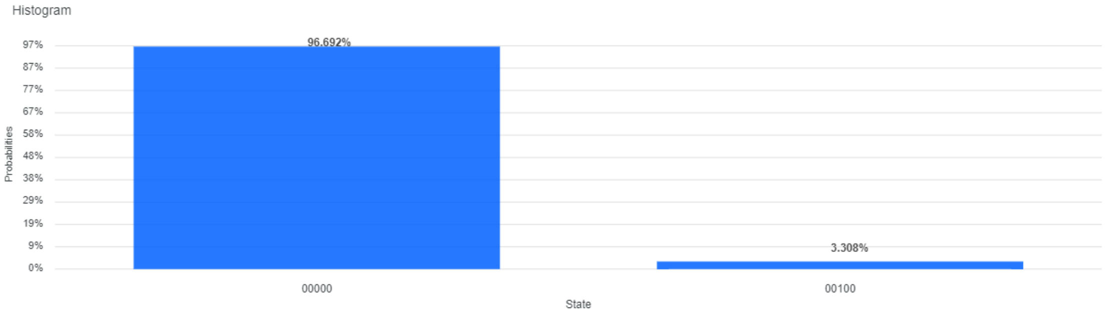

The excited state population is $p_e(3\delta\tau) = 0.03308$. The raw data was obtained on April 17th, 2020.

For the sub-channel choice ↓↓↑, the circuit and the result are

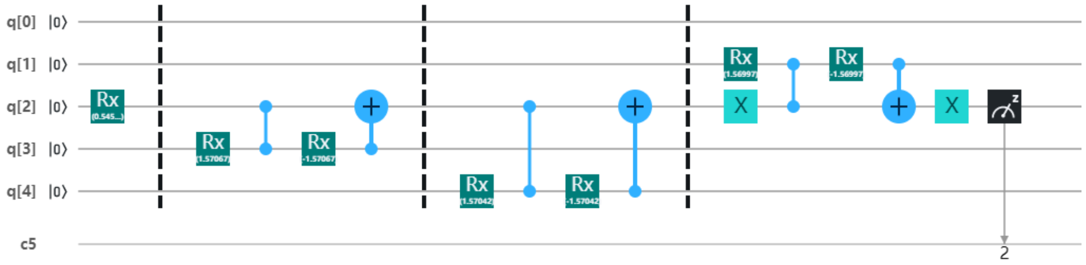

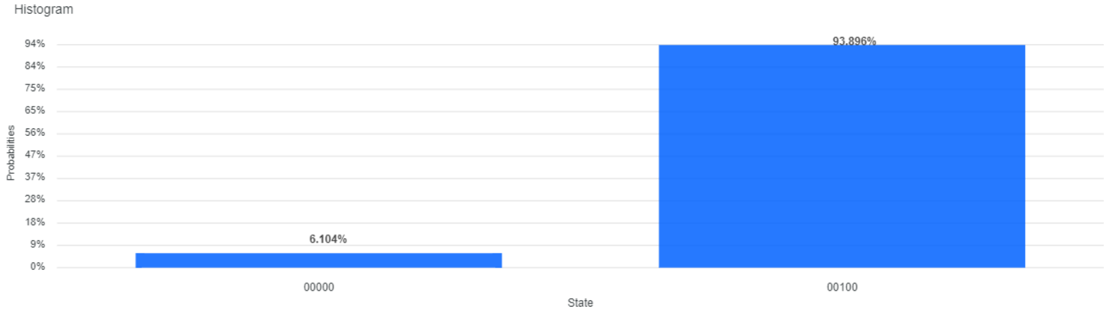

The excited state population is $p_e(3\delta\tau) = 0.93896$. The raw data was obtained on April 17th, 2020.

For the sub-channel choice ↓↓↓, the circuit and the result are

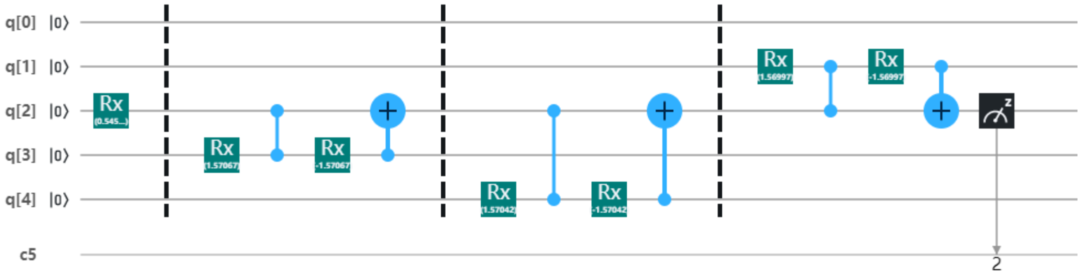

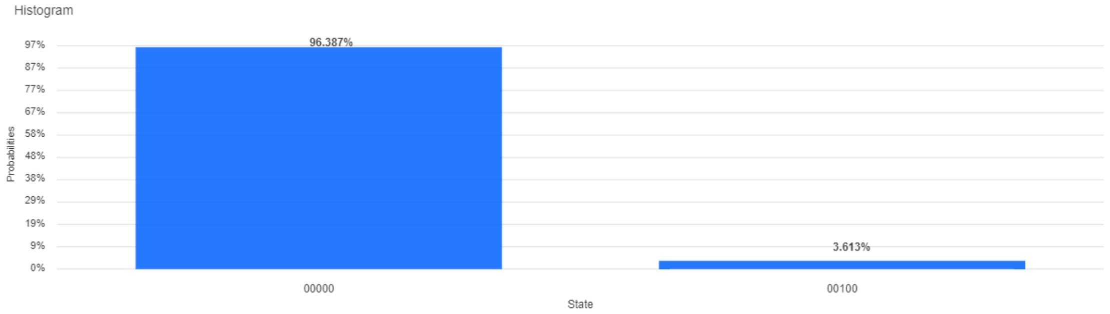

The excited state population is $p_e(3\delta\tau) = 0.03613$. The raw data was obtained on April 17th, 2020.
